# Advancements and prospects of CRISPR/Cas9 technologies for abiotic and biotic stresses in sugar beet

**DOI:** 10.3389/fgene.2023.1235855

**Published:** 2023-11-09

**Authors:** Varucha Misra, A. K. Mall, Himanshu Pandey, Santeshwari Srivastava, Avinash Sharma

**Affiliations:** ^1^ ICAR-Indian Institute of Sugarcane Research, Lucknow, India; ^2^ Khalsa College, Amritsar, India; ^3^ Faculty of Agricultural Sciences, Arunachal University of Studies, Namsai, India

**Keywords:** abiotic, biotic, CRISPR/Cas, genes, genome editing, tolerance

## Abstract

Sugar beet is a crop with high sucrose content, known for sugar production and recently being considered as an emerging raw material for bioethanol production. This crop is also utilized as cattle feed, mainly when animal green fodder is scarce. Bioethanol and hydrogen gas production from this crop is an essential source of clean energy. Environmental stresses (abiotic/biotic) severely affect the productivity of this crop. Over the past few decades, the molecular mechanisms of biotic and abiotic stress responses in sugar beet have been investigated using next-generation sequencing, gene editing/silencing, and over-expression approaches. This information can be efficiently utilized through CRISPR/Cas 9 technology to mitigate the effects of abiotic and biotic stresses in sugar beet cultivation. This review highlights the potential use of CRISPR/Cas 9 technology for abiotic and biotic stress management in sugar beet. Beet genes known to be involved in response to alkaline, cold, and heavy metal stresses can be precisely modified via CRISPR/Cas 9 technology for enhancing sugar beet’s resilience to abiotic stresses with minimal off-target effects. Similarly, CRISPR/Cas 9 technology can help generate insect-resistant sugar beet varieties by targeting susceptibility-related genes, whereas incorporating *Cry1Ab* and *Cry1C* genes may provide defense against lepidopteron insects. Overall, CRISPR/Cas 9 technology may help enhance sugar beet’s adaptability to challenging environments, ensuring sustainable, high-yield production.

## Graphical Abstract

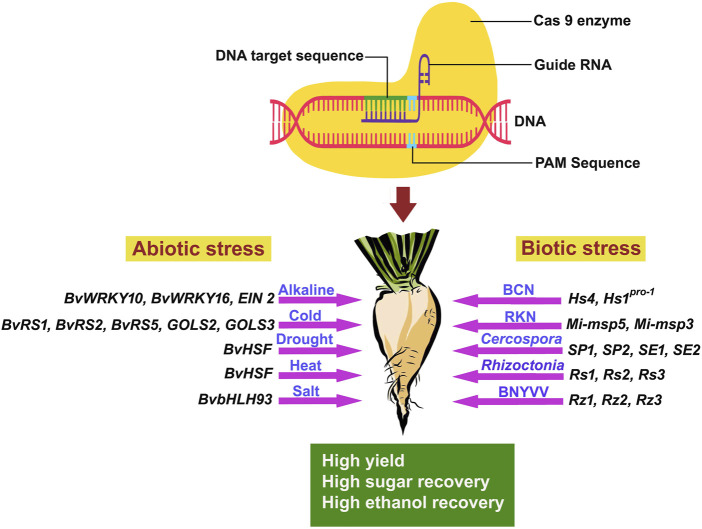

## 1 Introduction

Sugar beet (*Beta vulgaris* L.) is cultivated in temperate regions of the world, however, its cultivation has spread to the tropical and subtropical zones of India (Mall et al., 2022a; Misra et al., 2022a). Sugar beet is well known for its sugar production in many countries of the world covering a significant area under cultivation (Table 1) and contributing to around 30% of the world’s sugar requirement (Zicari et al., 2019). The root of sugar beet is an important source of natural sucrose as a sweetening agent (Mall et al., 2022b) and has diverse industrial applications (Misra et al., 2018). Sugar beet production faces major threats from biotic and abiotic stresses (Mulet, 2022). For instance, common viral (beet necrotic yellow vein virus, BNYVV (Ramchandran et al., 2021), bacterial (like *Pseudomonas aptata*), and fungal diseases (like *Cercospora* (Misra et al., 2022c); *Alternaria* (Misra et al., 2020a), as well as nematodes (*Meloidogyne incognita*), and insects (like *Aphis fabae* (Mulet, 2022), *Spodoptera litura* (Santeshwari et al., 2020; Baitha et al., 2022)) hampers the production and productivity of the crop. Salinity, drought, heat (high temperatures), and cold are some of the abiotic stresses that severely impact sugar beet production all over the world (Misra et al., 2020b) (Table 2). Besides, other abiotic stresses like ozone build-up, flooding, nutritional deficiency, and heavy metal poisoning of the soil can also be challenging (Shabbir et al., 2022). In order for sugar beet plants to produce their optimum amount of sugar, enough moisture availability on a daily basis is necessary so as to allow for efficient transpiration and photosynthesis processes (Ober and Rajabi, 2010; Barratt et al., 2023). However, such ideal circumstances under natural environmental conditions have not been observed frequently owing to scanty rainfall or limited irrigating options. Despite the concerted efforts in developing tolerant varieties of sugar beet through conventional breeding and genome editing for improving the sugar and ethanol yield (Pattanayak et al., 2023), the scope still exists in the field of development of sugar beet varieties tolerance/resistance to abiotic and/or biotic stresses.

**TABLE 1:** Sugar beet area, yield, and production in major sugar beet producing countries of the world.

Country	Area (ha)	Yield (100 gm/ha)	Production (t)	References
China	229,300	342,386	7,850,900	FAO (2022)
France	491,880	855,116	34,365,390	
Germany	390,700	817,645	31,945,400	
Iran	91,803	560,650	5,146,924.8	
Italy	27,910	541,279	1,510,710	
Poland	250,570	609,564	15,273,850	
Russia	993,830	414,575	412,016,686	
Turkiye	288,940	631,620	18,250,000	
Ukraine	226,600	478,989	10,853,880	
United States	448,230	743,813	33,339,950	

**TABLE 2:** Impact of abiotic/biotic stresses on sugar beet yield.

Stresses		Reduction in sugar beet root yield (%)	References
Abiotic	Salt stress	49.3	Anagholi et al. (2018)
		10–50	Bybordi (2010)
	Cold	77 (in dry matter reduction)	Jalilian et al. (2017)
	Drought	27 (50% less irrigation); 21 (50% less irrigation)	Ghaffari et al. (2022)
		5 (Northern Europe)	Pidgeon et al. (2001)
		30 (Southern Russia)	
Disease resistance			
Biotic	Beet cyst nematode (*Heterodera schachtii* Schmidt)	25 to 50	Agrios (2005)
		21 (Italy)	Greco et al. (1993)
		60	Grujicic (1958); Cooke (1987)
		70	Pylypenko and Kalatur (2015)
	*Cercospora* leaf spot	30	Tan et al. (2023)
		40	Smith and Ruppel (1973); Esh and Taghian (2022)
		20–25 (India)	
	Beet curly top virus (BCTV)	30	AgResearch Magazine (2016)
	Beet necrotic yellow vein virus (BNYVV)	90	Johansson (1985); Casarini (1999)
Insect Pests			
	Armyworm (*Spodoptera* spp.)	>25 (Foliage damage)	DiFonzo et al. (2006)

Molecular biology has witnessed a massive transformation due to the emergence and development of the CRISPR/Cas system as a biotechnological tool. The CRISPR/Cas 9 system is reportedly an efficient technology (Ahmad et al., 2021). The microbial adaptive immune system, CRISPR may target any genomic region by using a synthetic short guide RNA (sgRNA) (Jinek et al., 2012). Its strength comes in its capacity to effectively and precisely cause double-strand breaks in DNA at any location in the genome. CRISPR mediated genome editing can be used to change practically any sequence to expose its role in the genome (Asmamaw and Zawdie, 2021). On the basis of the genes encoding the effector modules CRISPR–Cas systems can be classified where different cas proteins have unique characteristics and functional roles (Chaudhuri et al., 2022) (Table 3). CRISPR/Cas 9 mediated genome editing requires a protospacer adjacent motif (PAM) for Cas nuclease for initiation of the cutting process. The PAM is located 3-4 nucleotides downstream from the specific site where cleavage needs to be done (Gleditzsch et al., 2019). The advances in genome editing techniques, particularly CRISPR/Cas 9 system, will benefit the cultivation of sugar beet by developing varieties resistant to abiotic and biotic stress conditions. This review discusses the current understanding of the mechanism of CRISPR/Cas 9 technology and its application in the improvement of sugar beet cultivars against abiotic/biotic stresses.

**TABLE 3:** Different types or classes of Cas proteins, emphasizing their unique characteristics and functional roles.

Protein	Class	Type	Sub type	Process	Functions
Cas 1	1, 2	I, II, III, IV, V, VI	A, B, C, D, E, F, U	Spacer acquisition	DNAse, bind RNA; Cleavage at the specific site; Allow insertion of new spacers into CRISPR arrays
Cas 2	1, 2	I, II, III, IV, V, VI			Specific to U-rich regions; Homologous to mRNA interferase
Cas 3 (Signature)	1	I		Target interference	DNA helicase endonuclease; Cutting the target DNA at specific sites due to endonuclease domain
Cas 4	1, 2	I, II		Spacer acquisition and regulation	RecB-like nuclease homologous to RecB; Exonuclease activity and binds with RecBCD
Cas 5	1	I		crRNA expression and target binding	RAMP protein, crRNA biogenesis; (small subunit protein) Catalysation of crRNA processing; binds to a large subunit of Cas 8 in type I and Cas 10 in type III
Cas 6	1	I, III			RAMP protein, crRNA biogenesis; (nuclease activity) Repeat specific RNase involves in crRNA processing
Cas 7	1	I			RAMP protein, crRNA biogenesis; Co-transcriptional RNA cleavage during interference
Cas 8	1	I			Large protein with McrA/HNH-nuclease domain; Homologue of Cas10 protein; large subunit binding occurs with cas5 subunit
Cas 9 (Signature)	2	II	A, B, C	crRNA Target interference	Large multidomain protein with McrA-HNH nuclease domain; (Type II signature protein) crRNA dependent nuclease; Mediates RNA-guided DNA cleavage; widely used as DNA nucleases for inducing site-specific DNA breaks
Cas 10 (Signature)	1	III	A, B, C, D	crRNA expression and interference	HD nuclease domain, palm domain, Zn ribbon; Genetic manipulation
Cas 12a	2	V	A	Spacer acquisition	Cleaves the both complementary strands of the targeted DNA segment using a single RuvC nuclease domain
Cas 12b	2	V	B	Target interference	Dual-RNA-guided DNA nuclease and requires tracrRNA for further processing; higher efficiency for gene activation; wider target site for gene suppression; prefers T-rich protospacer adjacent motifs (PAMs)
Cas 12c	2	V	C	crRNA expression	Target binding rather than target degradation
Cas 12d (Signature)	2	V	D	Target interference	Catalyzes DNA cleavage with short complementary untranslated RNA
Cas 12e (Signature)	2	V	E	Target interference	Target DNA unwinding
Cas 13	2	VI	A, B, C, D	crRNA expression	Interference activity; Degradation of mRNAs; impart tolerance against plant viruses

## 2 Site-directed nucleases (SDNs) and comparison of zinc finger nucleases, transcription activator like effector nucleases with CRISPR/Cas 9 technology

Targeted genome engineering has been emerged as an alternative to traditional plant breeding approaches, aiming to achieve a variety of crop improvement goals and sustainable food production (Misra et al., 2018). Genetic engineering (GE) techniques have been deployed to enhance the quality attributes of sugar beet (e.g., shelf life) (Monteiro et al., 2018) and improve its tolerance to biotic and abiotic stresses (Wan et al., 2021). In this context, the application of site-directed nucleases (SDNs) has evolved as a suitable GE technique for introducing desirable characteristics into plants (Aglawe et al., 2018). Zinc finger nucleases (ZFNs), transcription activator like (TAL) effector nucleases (TALENs), and Clustered regularly interspaced short palindromic repeats and CRISPR-associated (CRISPR/Cas) technology are instances of SDN methods. SDNs enable precise changes at predetermined places in a genome, avoiding any unintended random mutagenesis. They offer unparalleled control over targeted genome alterations, proving to be more cost-effective and efficient than conventional plant breeding and genetic engineering methods. SDNs have the potential to aid in crop improvement and enhance food security in many sugar beet producing countries (Table 4). SDNs are categorised into SDN1, SDN2, and SDN3 based on the outcomes of genomic alterations and double strand break repair (Ghouri et al., 2023). SDN1 does not require template and causes gene disruptions *via* InDels (small insertions or deletions of bases). SDN2 uses a homologous template to repair or modify the gene at one or more locations. SDN3 requires the use of a whole gene as a template and results in gene substitution or foreign DNA insertion (Chen and Gao, 2020).

**TABLE 4:** Legislation of different sugar beet producing countries for genome editing crops.

Countries	Legislation	SDN 1	SDN 2	SDN 3	Year approved	Crops approved	References
United States	Classified SDN-1,2 genome-edited crops are equivalent to traditional breeds	Deregulated		Case by case	2018	Corn	Schmidt et al. (2020); Herrera et al. (2017); Chaturvedi (2004); Lombardo and Grando. (2020)
						Tomato	
					2017	Soyabean	
					2016	Mushroom	
					2017	Flax	
China	The Ministry of Agriculture announced preliminary recommendations for evaluating the safety of genome-edited plants that do not include exogenous DNA.	Under development			Not Applicable	No crops approved	Mallapaty (2022); Haque et al. (2018); Chaturvedi (2004); Wang et al. (2015a)
India	According to the memorandum, working with genome-edited plants must be done with extreme caution until exogenous inserted DNA is no longer present. The guidelines apply to SDN-1 and SDN-2 genome-edited plants	Under development			Not Applicable	No approved crops	Buchholzer and Frommer (2023); Lombardo and Grando. (2020); Chaturvedi (2004)
Russia	According to Resolution of 22 April 2019 no. 479, provision of funding for genome editing and defined transgene-free modified crops as equivalent to those produced through conventional breeding	New polices are expected			Not Applicable	No approved crops	Dobrovidova (2019)
UK	Initially, genome-edited crops will be free from GMO field trial rules. Field testing defines “qualified higher plants” as genome-edited plants that might have been developed using standard breeding procedures or may have occurred naturally. It is predicted that genome-edited plants will then be able to successfully enter field trials and acquire commercial approval without the need for case-by-case review	Case by case			Not Applicable	No approved crops	Stokstad (2021)
Brazil	Products obtained through site-directed random mutation involving the joining of non-homologous ends (SDN1 mutation) or site-directed homologous repair involving one or few nucleotides (SDN2 mutation) meet the criteria established in Normative Resolution No. 16 to be designated as non-GMO on a case-by-case basis. according to the resolution’s provisions, site-directed transgene insertions (SDN3 mutation) are classified GM. If the product is labelled as GMO, the developer must meet all biosafety regulations and will be approved only after the CTNBio risk assessment. If the product is labelled non-GMO, it can be registered using the same methods as conventional items	Deregulated		Deregulated (If not transgenic)	NA	No approved crops	Schiemann et al. (2020); Chaturvedi (2004); Lombardo and Grando. (2020)

The CRISPR/Cas system is a convenient replacement for ZFNs and TALENs in generating targeted genomic alterations (Ahmad et al., 2021). Both ZFNs and TALENs are utilized to mutate genomes at specific loci (Boti et al., 2023). However, these systems require two distinct DNA binding proteins flanking the region of interest, each having a C-terminal *Fok*I nuclease module. Custom proteins are necessary for targeting DNA sequences. The process of designing and constructing custom proteins in both these technologies (Zinc finger motifs in ZFNs, while DNA binding domains obtained from TALE in TALENs, are required) is laborious and time-consuming. In CRISPR-mediated genome editing, PAM is located 3-4 nucleotides downstream from the specific site where cleavage will occur (Gleditzsch et al., 2019). Therefore, accessibility to the protospacer adjacent motif (PAM) site, as the main determining factor for altering any functioning sequence, is easier (Akram et al., 2023). CRISPR technology depends on RNA-guided sequences, which can be designed effortlessly (Bajpai et al., 2023).

The target specificity in ZFNs and TALENs is more challenging as zinc fingers or TALEs identify short DNA sequences and require the combination of multiple modules to target the desired region, which may result in off-targets (Jyoti et al., 2023). The probability of off-targets is very low in CRISPR because it utilizes guide RNA to target the desired site, which can be easily programmed. Furthermore, the application of custom protein engineering in ZFNs and TALENs causes lesser adaptation and more laborious efforts (Kalaitzandonakes et al., 2022) while altering in sgRNA is easier for targeting the desired gene in CRISPR technology. The use of sgRNA in CRISPR technology is also beneficial in the delivery stage, making it a simple process compared to ZFNs and TALENs (Pankaj and Kumar, 2023). Due to the complexity involved in ZFNs and TALENs, including design and construction, target specificity, flexibility, and delivery), these methods are not preferred compared to CRISPR (Bhatia et al., 2023).

## 3 Application of CRISPR/Cas 9 technology for abiotic stress resistance in sugar beet

Sugar beet (*Beta vulgaris* L.) production is drastically affected by biotic and abiotic factors, which reduce the rate of photosynthesis, expansion of the canopy, development of the root system, and consequently, the accumulation of sucrose content in the plant (Misra et al., 2022a). Abiotic stresses, including temperature fluctuations, water scarcity, salinity, metal toxicity, and UV radiation, are harmful to the sugar beet crop, and severely affect its yield across the world (Yu et al., 2020). These stressors greatly restrict the distribution of sugar beet crops, affect their developmental processes, and decrease sugar beet productivity (Ober and Rajabi, 2010). Improved understanding of multiple molecular mechanisms, like pathway signalling, activation of transcription factors, transcript modification (post-transcriptional modification), translation of processed transcript, and protein modifications after the translation process, underlying stress responses of sugar beet crops at multiple levels, would be helpful in increasing the sugar beet production and sustainability through the application of CRISPR/Cas 9 (Yu et al., 2020; Misra et al., 2022a).

Abiotic stress tolerance is a complex trait that is mediated by multiple genes. The components of metabolic, regulatory, and signalling networks in plants interact and crosstalk extensively under abiotic stress conditions (Garg et al., 2014; Mickelbart et al., 2015). Several abiotic stress resistance-conferring genes have been identified in plants (Table 5) and introgressed into related crops through the application of biotechnological tools (Razzaq et al., 2021).

**TABLE 5:** Identified genes in other crops for providing abiotic stress tolerance through CRISPR/Cas technology.

Abiotic stress	Genes targeted	Crops	References
Alkaline stress	*OsPPa6*	*Oryza sativa*	Wang et al. (2019)
Cold stress	*OsPIN5b, GS3, OsMYB30, OsAnn5, OsAnn3, OsPRP1*	*Oryza sativa*	Zeng et al. (2020), Shen et al. (2017),
			Nawaz et al. (2019)
	*AtWRKY34*	*Arabidopsis thaliana*	Zou et al. (2010)
	*SlCBF1*	*Solanum lycopersicum*	Li et al. (2018)
	*VvWRKY24*	*Vitis vinifera*	Wang et al. (2014)
	*BcWRKY46*	*Brassica campestris*	Wang et al. (2011)
Heat stress	*OsPDS, OsHSA1, OsNAC006, OsNA C006, OsPyl14/6*	*Oryza sativa*	Nandy et al. (2019), Qiu et al. (2018), Wang et al. (2020), Miao et al. (2018)
	*AtWRKY25/26, AtWRKY33, AtWRKY39*	*Arabidopsis thaliana*	Li et al. (2011), Jiang et al. (2008), Park et al. (2005)
Drought stress	*AtOST2, AtAREB1, AtAVP1, AtmiR169a*	*Arabidopsis thaliana*	Osakabe et al. (2016), Roca Paixão et al. (2019), Park et al. (2017), Zhao et al. (2016)
	*OsERA1, OsSAPK2, OsSRL1, OsSRL2, OsDST, OsNAC14, OsPUB67*	*Oryza sativa*	Ogata et al. (2020), Lou et al. (2017), Liebe et al. (2020), Santosh Kumar et al. (2020)
	*BnaA6.RGA*	*Brassica napus*	Wu et al. (2020)
	*BdWRKY36*	*Brachypodium distachyon*	Sun et al. (2014)
	*BcWRKY46, BnaA6.RGA*	*Brassica campestris*	Wang et al. (2011), Wu et al. (2020)
	*GmMYB118*	*Soyabean*	Du et al. (2018)
	*GmMYB118*	*Chickpea*	Badhan et al. (2021)
Salt stress	*AtWRKY, AtWRKY4, AtACQOS*	*Arabidopsis thaliana*	Li et al. (2021a), Kim et al. (2021)
	*OsDST, OsSPL10, OsRAV2, OsBBS1, OsNAC45, OsAGO2, OsVDE, OsRR22, OsSAPK2, OsPQT3, OsPIL14, OsBGE3*	*Oryza sativa*	Santosh Kumar et al. (2020), Lan et al. (2019), Duan et al. (2016), Zeng et al. (2018), Zhang et al. (2020b), Yin et al. (2020), Wang et al. (2003), Zhang et al. (2019), Mo et al. (2020), Alfatih et al. (2020), Yin et al. (2017)
	*VpWRKY1, VpWRKY2, VpWRKY3*	*Vitis pseudoreticulata*	Li et al. (2010), Zhu et al. (2012)
Herbicide stress	*OsTB1, OsALS, OsACC*	*Oryza sativa*	Butt et al. (2018), Zhang et al. (2020b), Lyu et al. (2022)
Metal stress	*Atoxp1*	*Arabidopsis thaliana*	Baeg et al. (2021)
	*OsARM1, OsNramp5, OsLCT1, OsHAK1, OsPRX2*	*Oryza sativa*	Wang et al. (2017b), Tang et al. (2017), Lu et al. (2017), Nieves-Cordones et al. (2017), Mao et al. (2019)

Gene activation or repression occurs by targeting transcriptional activator or repressor complexes to particular sites within the gene promoter region with catalytically inactivated Cas endonuclease. For this purpose, CRISPR-based approaches like CRISPRa and CRISPRi could be beneficial in characterizing genes for abiotic stress tolerance (Osakabe et al., 2016). CRISPR activation, abbreviated as CRISPRa, is a CRISPR variation in which a catalytically dead (d) Cas 9 is coupled with a transcriptional effector to control target gene expression. When the guide RNA and the effector arm reach the genomic location, the dCas9 is unable to produce a cut, and the effector instead triggers downstream gene expression. CRISPRa technique is used to increase gene expression by targeting the promotor region upstream of the transcription start site (TSS), whereas the CRISPR interference (CRISPRi) approach suppresses gene expression by targeting the promotor region downstream of the TSS. The application of CRISPRa/i requires precise identification of the TSS location (Davis et al., 2018; Thomas et al., 2019). CRISPRi is a technology that uses dCas9’s programmable binding capacity to inhibit gene expression by preventing or interfering with RNA polymerase binding, transcription factor binding, and transcriptional elongation. sgRNA specific to a gene sequence’s upstream regulatory region (e.g., promoter) or transcription initiation site could direct dCas9 to bind and inhibit transcription initiation or elongation, effectively silencing gene expression. To inhibit target gene expression, dCas9 is coupled with the transcription repression domain of the Kruppel associated box (KRAB). dCas9 alone or in combination with KRAB is an effective tool for knocking off one or more genes (Piatek et al., 2015). CRISPRi and CRISPRa regulate gene expression by increasing or suppressing RNA polymerase, respectively. Thus, CRISPRi and CRISPRa are promising approaches for exploring and regulating stress-regulatory genes, as well as developing abiotic stress tolerant varieties. CRISPRi/a has been utilized successfully in plants to modify expression by a factor of 1000 (La Russa and Qi, 2015). CRISPRi and CRISPRa technologies can also be used in sugar beet under abiotic stress conditions. Although there is a lack of information available on this aspect, there are prospects to be worked on. The application of these technologies can aid in regulating gene expression in response to abiotic stress conditions (McCarty et al., 2020). These could be correlated with gene expression in sugar beet under drought, salinity, or temperature stress to provide tolerance. Altering these gene expressions could produce resilient sugar beet varieties with respect to abiotic stress conditions. Inhibiting or activating the targeted stress-responsive genes in sugar beet under abiotic stress conditions will help in understanding the regulatory mechanism. This, in turn, will assist in developing sugar beet varieties tolerant to adverse environmental conditions.

Furthermore, Cas 12a could also be explored for targeting the specific genes contributing to abiotic stress pathways, like drought, salinity, temperature, etc. By altering the genes related to specific abiotic stress conditions in sugar beet, the plant’s potential to adapt and survive under adverse conditions could be increased. For instance, Chen X. et al. (2018) reported the application of Cas12a in targeting drought-responsive genes (positive regulation of gene) in *Arabidopsis*. Altering these targeted drought-responsive genes through Cas 12a improved the plant’s drought tolerance potential. Such plants exhibited better water retention capacity, reduction in wilting, and higher survivability under drought conditions.

**FIGURE 1:**
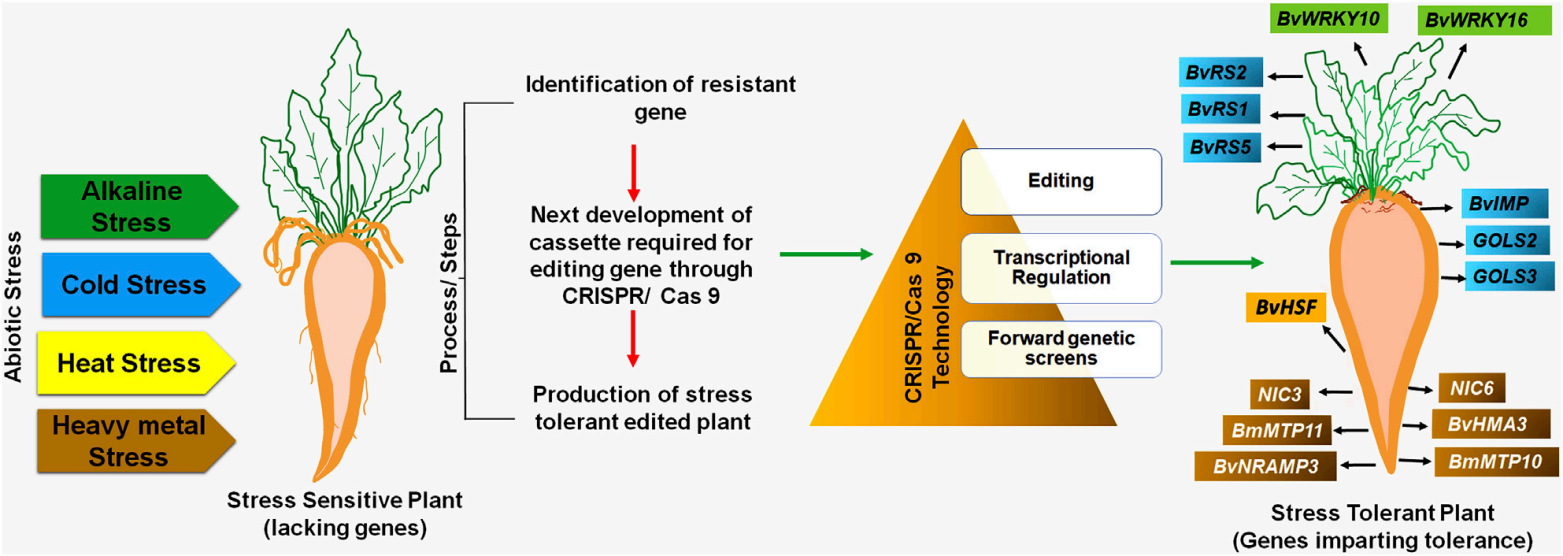
CRISPR/Cas 9 mechanism for abiotic stress tolerance in sugar beet.

The development and evolution of sugar beet varieties tolerant to abiotic stress induced by climate change and global warming are imperative (Yolcu et al., 2021). Therefore, CRISPR/Cas 9 technology is necessary to create highly resistant sugar beet varieties against biotic and abiotic stresses. The CRISPR/Cas 9 technology involves specific steps for developing novel sugar beet varieties for different abiotic stress tolerances (Figure 1).

### 3.1 Alkaline stress tolerance

Aside from ionic toxicity and osmotic stress, high pH in the alkaline soils disrupts cell pH stability, destroys cell membrane integrity, and reduces root vitality and photosynthetic activity (Fang et al., 2021). Under the condition of high saline stress, beet varieties that are cultivated along with wild types (naturally occurring/non-domesticated beet varieties) exhibited high antioxidant enzyme activities (Wang et al., 2017; Li B. et al., 2021). Wu et al. (2019) reported that with increasing sodium bicarbonate concentrations, Na^+^ concentrations were enhanced significantly in shoots and roots of sugar beet plants under alkaline conditions while a steady level of potassium ion concentrations was observed. Maintenance of K^+^ and Na^+^ homeostasis could be a key strategy for sugar beets adjusting to alkaline stress. Several genes (like WRKY, NAC, MYB, etc.) have been reported to be involved in conferring abiotic stress tolerance in plants (Chinnusamy et al., 2006; Hennig, 2012; Khadiza et al., 2017).

Wu et al. (2019) reported 58 *WRKY* genes, and among them, 9 genes were found to be responsible for alkaline stress responses (∼15 mM–100 mM NaCHO_3_) in both shoot and root parts. It evidently proved the increased expression of the *BvWRKY10* gene (in the terminal and lateral shoots) and *BvWRKY16* gene (in roots) under alkaline stress. WRKY proteins have been known to be associated with the response of plants to biotic and abiotic stress conditions (Jiang et al., 2017). The quantitative alterations and tissue-specific expressions of different *BvWRKY* genes have clearly shown its implications for alkaline stress tolerance in sugar beet. Research to alter the expression of *BvWRKY* genes using CRISPR/Cas 9 technology is required for the development of stress-resistant varieties. The *BvWRKY* family genes are specific and play crucial roles in wider aspects of sugar beet development processes like germination, root development, photosynthesis, etc., including response to alkaline stress conditions (Wu et al., 2019). Therefore, these genes can be engineered through CRISPR/Cas 9 technology to develop alkaline stress-resistant varieties in sugar beet (Table 6). Furthermore, long noncoding RNAs (lncRNAs) in response to alkaline stress had also been identified and characterized in sugar beet leaves. Besides, the interactions of candidate genes and miRNAs with the lncRNAs under stress conditions have also been reported in sugar beet (Zou et al., 2020). The use of CRISPR/Cas 9 technology on these identified/characterized lncRNAs will be helpful in evaluating their function in sugar beet and its expression can be modified to provide alkali stress tolerance in sugar beet. Additionally, genes belonging to the bHLH (basic helix–loop–helix) family in sugar beet involved in salt stress tolerance have also been well known. Wang Y. et al. (2021) reported the *BvbHLH93* gene as a salt-responsive gene that has been shown to confer tolerance under salt stress conditions in sugar beet. By increasing antioxidant activity and decreasing ROS generation, the *BvbHLH93* gene modulates salt stress tolerance in sugar beets. Furthermore, the *BvbHLH93* gene’s ability to reduce *RbohD* and *RbohF* gene expression through modulating polyamine metabolism warrants additional investigation in sugar beet. CRISPR/Cas 9 technology will help in understanding the role and expression of these genes in salt stress conditions. This will aid in knowing the regulation of this gene in conferring tolerance to sugar beet for salt stress conditions.

**TABLE 6:** Transcription factor that can be engineered through CRISPR/Cas 9 in sugar beet to provide resistance to different abiotic stress conditions.

Abiotic stresses	Prospective transcription factor that can be targeted for CRISPR/Cas 9	Gene function	Gene expression	Editing mechanism	References
Alkaline	*BvWRKY10*	Provides resistance to alkaline and saline stress	Upregulation	Prime and base editing	Wu et al. (2019)
	*BvWRKY16*				Li et al. (2020a)
	*EIN 2*	• Ethylene insensitive protein	Induce/Increase	Prime and base editing	Lei et al. (2020)
		• Regulates ethylene response			Zhang et al. (2008)
		• Provides tolerance even against green peach aphid			Zhou et al. (2020)
Salt	*BvbHLH93*	• Provides resistance to salt stress	High expression	Prime and base editing	Wang et al. (2021a); Wang et al. (2021b)
		• Modulates salt stress tolerance			
		• Reduces *RbohD* and *RbohF* gene expression			
Chilling/Cold stress	*BvRS1*	• Stress genes responsible for raffinose synthase	Induce	Prime and base editing	Kito et al. (2018)
		• Provide resistance			
		• Encodes protein (comprises 783 amino acids)			
	*GOLS2*	• Genes responsible for galactinol synthase	Upregulation	Prime and base editing	Keller et al. (2021)
		• Provide resistance			
	*GOLS3*	• Stimulated by cold stress	Upregulation	Prime and base editing	
		• Provide resistance			
Heat	*BvHSF*	• Genes responsible for higher expression of heat shock proteins during elevated temperature	Upregulation	Prime and base editing	Ismail et al. (2020)
		• Provide resistance			

### 3.2 Cold stress tolerance

Chilling temperature affects sugar beet cultivation, production, and economic yield (Moliterni et al., 2015; Bhattarcharya, 2022). Cold stress conditions have a serious impact on the sugar beets at various developmental stages, including early germination, sugar metabolism, growth, and bolting in the roots (Hoffmann and Kluge-Severin, 2011). The seedling stage of sugar beet is particularly prone to low temperature stresses. Cold stress during this stage leads to severe degeneration and growth retardation in the root system, consequently decreasing its sugar content (Moliterni et al., 2015; Jalilian et al., 2017). Porcel et al. (2018) revealed that overexpression of *BvCOLD1* gene in sugar beet exhibits cold tolerance potential along with other abiotic stress tolerance and overcoming boron deficiency. Keller et al. (2021) found that improved freezing-tolerant sugar beet genotypes accumulated more raffinose in the pith portion, which is vulnerable tissue to freeze damage. This finding demonstrated that raffinose and its precursors protect sugar beets from freezing damage. Recognizing the importance of raffinose in providing cold tolerance to sugar beet plants, Kito et al. (2018) isolated and characterized two genes, *BvRS1* and *BvRS2,* from the sugar beet plant. These genes code for the expression of raffinose synthase, which is a crucial enzyme during raffinose biosynthesis. An increase in transcription levels of *BvRS1* and *BvRS2* genes was observed in response to chilling stress in both leaves and roots (Kito et al., 2018). Furthermore, the production and build-up of oligosaccharides belonging to the raffinose family are particularly important during cold hardiness. Galactinol synthase (GolS) is considered a key regulator of the synthesis of such oligosaccharides and their accumulation (Vinson et al., 2020). The expression levels of *GOLS2* and *GOLS3* genes, responsible for galactinol synthase, as well as *BvRS2* and *BvRS5,* were high during chilling temperatures (Keller et al., 2021). Interestingly, the product of the *BvRS5* gene product and raffinose content increased exceptionally during freezing temperature in the taproots of tolerant sugar beet varieties, GT2 and GT3. In comparison with other sugar beet germplasm, GT2 exhibited high expression of *GOLS* and *RS* genes and raffinose content in roots, indicating chilling resistance in GT2 (Keller et al., 2021; Yolcu et al., 2021). Membrane proteins also frequently recognize cold stress conditions in sugar beet. These proteins activate a Ca^2+^ signal in the cytosol. Ca^2+^-binding proteins may act as a bridge between the Ca^2+^ signal and several downstream transcription factors (Iqbal et al., 2022). It was reported that *B. vulgaris Integral Membrane Protein* gene resembles *AtERDL6*, which was previously reported for cold tolerance (Qi et al., 1995). Freezing conditions may be responsible for the increased transcription rate of *BvIMP* gene and vacuolar carbohydrates trafficking in sugar beet leaves, crucial for chilling stress response and germination of seed (Yu et al., 2020; Reyer et al., 2021).

Utilizing CRISPR/Cas technology, scientists have successfully enhanced cold stress tolerance in plants by editing genes allied with cold stress and raffinose synthesis. In the case of *Vitis vinifera*, the *VaDof17d* gene plays a critical role in the cold-responsive pathway and the production of raffinose family oligosaccharides. This is evidenced by the enhanced expression of galactinol synthase (GolS) and raffinose synthase genes. Mutating the *Dof17d*-*ED* gene through CRISPR/Cas 9 technology resulted in reduced cold tolerance and reduced levels of raffinose family oligosaccharides during cold stress (Wang et al., 2021). Therefore, genes including *BvIMP, BvRS1*, and *BvRS2* (Table 6) can be engineered in sugar beet through CRISPR/Cas 9 technology to develop site-specific mutants. These mutants could be instrumental in enhancing cold tolerance in sugar beet crops by boosting their expression.

### 3.3 Heat stress tolerance

Increased temperatures and water scarcity tend to drastically affect the water content in plants where excess transpiration decreases the rate of water intake and causes permanent wilting in the plant (Kumar et al., 2004). Sugar beet is greatly affected by altered climatic conditions and disturbances in weather (Abou-Elwafa et al., 2020). High temperatures hamper major metabolic activities such as germination of seed, seed viability and its vigor, etc., leading to a threat to the survival of crop plants (Stevanato et al., 2019). Critical physiochemical processes, including photosynthesis and photosystem (PSII) activity also greatly affected due to blockage in the electron transport chain under high temperature stress (Murakami et al., 2000; Moore et al., 2021). The recent expansion of sugar beet cultivation in tropic and sub-tropical regions has drawn attention to farming it during the summer season (Abou-Elwafa et al., 2020).

Recognizing the importance of sugar beet cultivation across the world during the summer season, Ismail et al. (2020) explored the role of the transcription factor *BvHSF* gene, which showed higher expression levels under heat stress. The sugar beet crop showed enhanced expression levels in response to water scarcity, heat stress, and drought stress. Heat shock transcription factors (HSFs) are pivotal transcription factors in plants, critical for their response to various abiotic stresses such as heat, cold, salt, and drought (Fan et al., 2021). These HSF family members act by binding to the reverse repeat region of heat shock elements (HSEs), facilitating the transcription of heat shock proteins (HSPs) and assisting in the plant’s stress adaptation mechanisms (Guo et al., 2016; Jacob et al., 2017). Wu et al. (2022) demonstrated that CRISPR/Cas 9 knockout mutants, specifically targeting single copy *MpHSF* genes (*Mphsfa1V* and *Mphsfb1* mutants), resulted in different indel editing sites, showcasing enhanced thermotolerance in plants. Therefore, *BvHSF* genes can be engineered in sugar beet through CRISPR/Cas 9 technology to enhance their expression in sugar beet crops for increased tolerance towards heat resistance by creating in-dels (Table 6).

### 3.4 Drought stress tolerance

Drought stress also negatively impacts sugar beet root growth and development during the early phases of growth. Furthermore, the introduction of drought stress later in the growing season reduces leaf area and the number of leaves, ultimately resulting in lowered photosynthetic efficiency (Abou-Elwafa et al., 2020).

Generally, an increase in the expression of multiple drought-responsive genes and transcription factors enhances the plant’s ability to tolerate drought conditions (Fang and Xiong, 2015; Kumar et al., 2020; Santosh Kumar et al., 2020). Conversely, upregulation of drought-sensitive genes in plants heightens their vulnerability to drought stress due to imbalances in hormonal levels, reduced antioxidant activities, and heightened production of reactive oxygen species (ROS). CRISPR/Cas 9 based genome editing provides a promising avenue for enhancing drought tolerance in plants. This technique involves targeting negative regulators or drought-sensitive genes, allowing scientists to modify specific genetic elements and create crops that are more resilient to water scarcity. By precisely altering these genes, drought-resistant varieties can potentially be developed, ensuring sustainable agriculture in the face of changing environmental conditions. *WRKY* transcription factors are pivotal regulators of plant growth, development, and responses to both biotic and abiotic stresses. Among these factors, *WRKY3* and *WRKY4* genes in plants play a significant role in orchestrating the defense mechanisms against drought stress (Li B. et al., 2021). For instance, genetic manipulation of the *OsWRKY5* transcription factor has revealed significant insights into drought tolerance in plants. *OsWRKY5*, a key regulator, was found to hinder the plant’s ability to withstand drought. During the seedling and heading phases, *OsWRKY5* was primarily expressed in growing leaves, and its expression decreased under drought stress conditions. Researchers conducted experiments using genome-edited loss-of-function alleles, *oswrky5-2* and *oswrky5-3*, to enhance drought tolerance. These edited alleles resulted in increased drought resistance, as evidenced by improved plant growth even under water scarcity (Lim et al., 2022). Conversely, when *OsWRKY5* was overexpressed in the activation-tagged line *oswrky5-D*, plants exhibited greater susceptibility to drought stress. Overexpression of *OsWRKY5* led to heightened sensitivity to abscisic acid (ABA), a plant hormone involved in stress response, and encouraged ABA-dependent stomatal closure. By editing the *OsWRKY5* genome, researchers successfully enhanced the plant’s ability to produce grains even under drought stress conditions. This breakthrough offers valuable insights into improving crop resilience against water shortage, a critical factor in agricultural sustainability. Another example is for obtaining drought tolerance through CRISPR technology is enhancing the expression of *AREB1*, a specific transcription factor. In contrast, plants with a knocked-out *AREB1* gene exhibit increased sensitivity to drought stress (Singh et al., 2016). Roca Paixão et al. (2019) demonstrated enhanced drought stress tolerance through the utilization of CRISPR/dCas9 fusion with a Histone Acetyl Transferase (*AtHAT*) gene in *Arabidopsis*. These genes and transcription factors could also be targeted in sugar beet crops for attaining drought stress tolerance.

### 3.5 Heavy metal stress tolerance

Exposure of plants to toxic heavy metals causes different metabolic and physiochemical changes that depend on the concentration of these metals in soil, plant species, varieties, and abiotic conditions (Jamla et al., 2021; Thakur et al., 2022). Toxic metals like Pb damage the vacuolar membrane of sugar beet roots (Trela et al., 2012; Beata et al., 2022). Pb is one of the most toxic metals for plant cells, and it has a negative effect on the growth of plants, photosynthesis, respiration, and electron transport chain (Sharma and Dubey, 2005). Cd stress in *B. vulgaris* caused retarded growth, chlorosis, and increased root/plant ratio along with a decline in the rate of respiration in root-tips and photosynthesis (Greger and ögren, 1991; Larbi et al., 2002; Liu et al., 2022). Haque et al. (2021) observed that higher levels of Cd in sugar beet plants cause growth retardation due to an insufficient amount of Fe, resulting in decreased photosynthetic activity, and oxidative stress occurring in cells. Cd-treated plants display sensitivity to oxidative stress, leading to an increase in levels of O_2_^−^ and H_2_O_2_ in roots and shoots. Additionally, Haque et al. (2021) reported the antioxidant defense mechanism in sugar beet under a higher concentration of toxic metal and observed that Cd stress enhances the activity of catalase (CAT) enzyme in the shoots, whereas the activities of superoxide dismutase (SOD), ascorbate peroxidase (APX), and glutathione reductase (GR) do not increase either in roots or in shoots. Genes involved in heavy metal stress tolerance in sugar beets have been explored and proven to be important. There are two MTP genes, *BmMTP10* and *BmMTP11,* reported for metal-resistant proteins from wild species of sugar beet (*B. maritima*). The detoxification process of Ni was controlled by genes from wild sugar beet (*B. maritim*a) named as toxic nickel concentration (NIC), i.e., *NIC3*, *NIC6*, and *NIC8* (Bozdag et al., 2014; Yolcu et al., 2021). It is estimated that all these genes are required for protection against Ni toxicity. In a similar study, under Cd toxicity, sugar beet roots showed higher expression of putative *BvHMA3* and *BvNRAMP3* genes, suggesting that these genes are involved in the Cd uptake process (Haque et al., 2021).

Heavy metal-associated proteins (HMPs) and natural resistance-associated macrophage proteins (Nramp) are vital for heavy metal transport and detoxification within plant cells (Singh et al., 2016; Li W. et al., 2020). In rice, essential transporter genes such as *OsLCT1* and *OsNramp5* have been identified as key players in the absorption of Cd by the roots (Chang et al., 2020). Through CRISPR/Cas 9 enabled gene-expression manipulation, significant strides have been made in reducing the levels of Cd and Pb in rice grains. Specifically, the knockout of *OsNRAMP1* using CRISPR/Cas9 technology, as demonstrated in studies by Chu et al. (2022) and Wang F. Z. et al. (2017), has led to a substantial decrease in Cd and Pb content. Therefore, *BvHMA3* and *BvNRAMP3* genes can also be engineered in sugar beet through CRISPR/Cas 9 technology to develop heavy metal resistant varieties in sugar beet by creating site-specific mutagenesis.

## 4 Application of CRISPR/Cas 9 technology for improving biotic stress resistance

### 4.1 Pathogen resistance mechanism

**FIGURE 2:**
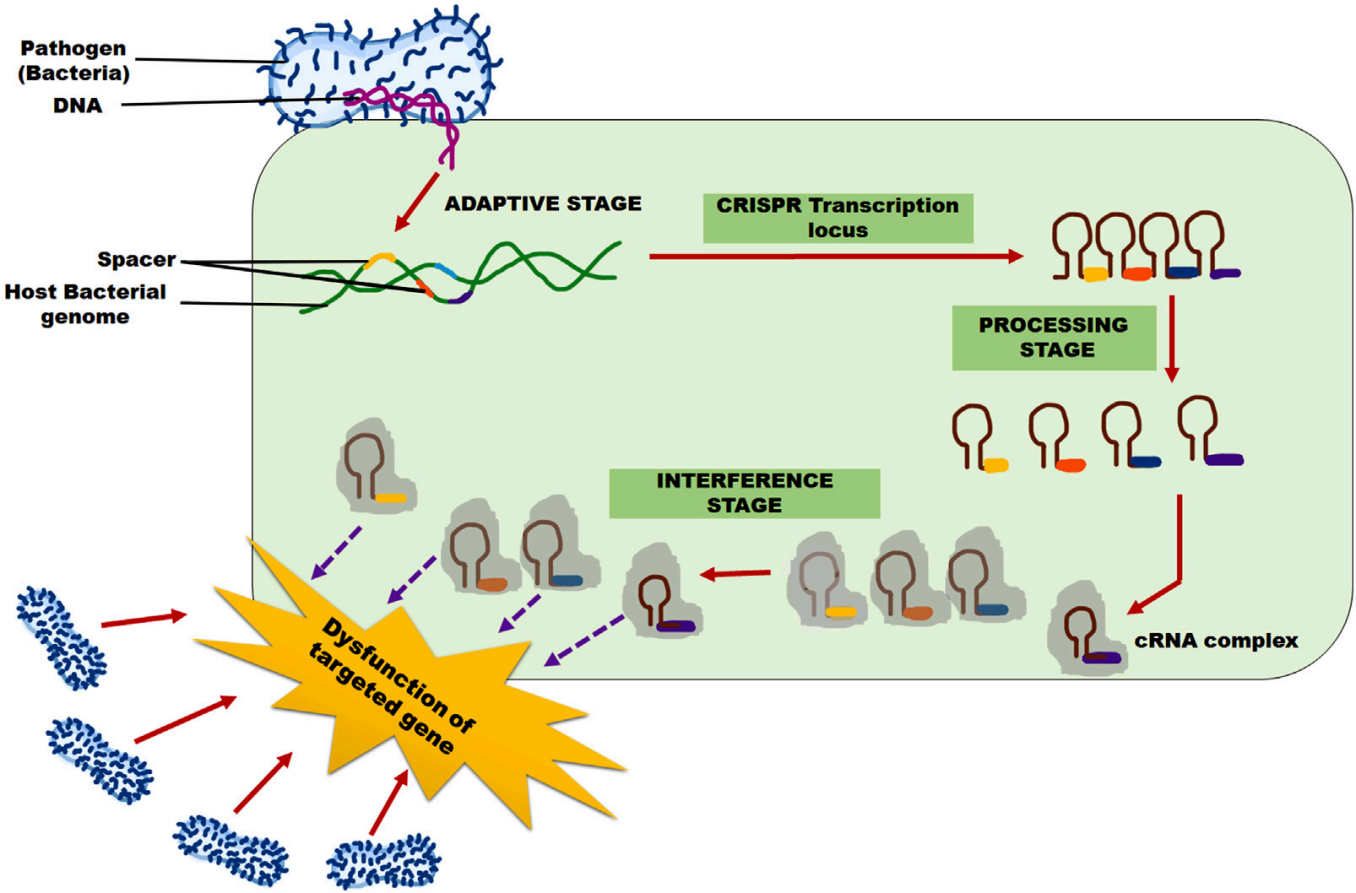
CRISPR/Cas 9 system mechanism for pathogen (Bacterial/Fungal/Viral) resistance in the plants. Adaptive, processing, and interference stages are three steps involved in this system.

The CRISPR/Cas 9 adaptive immune system for viral, bacterial, fungal resistance operates in three steps (Figure 2), regardless of the various shapes: 1) adaptation, 2) expression and maturity, and 3) interference. Protospacers, unique short DNA snippets from the invasive pathogen, are recognized by the Cas protein and inserted into CRISPR repeats as new spacers during the adaptation process. The host can then establish immunological memory and be equipped to recognize the same invasive infections in the future concerning this new spacer, which also serves as a genetic record (Paul et al., 2021).

For some CRISPR/Cas 9 systems to acquire the protospacer, the target DNA must have a short PAM (of 3-5 nucleotides) (Bolotin et al., 2005; Deveau et al., 2008; Shah et al., 2013). The CRISPR array is translated into a precursor-CRISPR RNA (pre-RNA), which is then processed to produce short mature CRISPR RNA (crRNA) through endo-nucleolytic cleavage and contains the sequences of the invading pathogen that have been learned (Carte et al., 2008; Haurwitz et al., 2010). At its 5′ end, each crRNA has a single spacer (a brief RNA segment that complements the DNA sequence of the foreign genetic material), and at its 3’ end, it has a CRISPR repeat sequence.

An active Cas-crRNA effector complex is formed when the mature crRNA combines with one or more Cas effector proteins. This complex searches for and attacks the cell’s foreign nucleic acids during the interference phase. Using the specific PAM sequence either upstream or downstream of the protospacer, and relying on Watson-Crick base pairing, the crRNA component of the complex serves as a guide to identify the target DNA (Semenova et al., 2011; Jiang et al., 2013; Westra et al., 2013; Fineran et al., 2014; Zetsche et al., 2015). Once the target DNA is successfully recognized, the Cas nuclease cleaves and digests it. Different classes of the CRISPR immune system, based on various effector Cas proteins and PAM recognition sequences, have emerged in recent years.

Invading foreign DNA fragments of virus particles are recognized and eliminated by the CRISPR/Cas 9 system, allowing it to identify and remove DNA or RNA sequences that facilitate continued invasion (Barakate and Stephens, 2016). CRISPR/Cas 9 technology modifies the plant’s inherent defense mechanism by detecting and removing harmful genes hidden within plant viruses. It can also be utilized to develop agricultural cultivars that are more resistant to specific plant viruses. This approach has fundamentally transformed virus resistance research because of its ability to use sequence-specific nucleases (Hsu et al., 2014).

### 4.2 CRISPR tools for disease diagnosis in sugar beet

The efficient implementation of control measures or management strategies heavily relies on the correct and timely identification of diseases and causative organisms. Consequently, disease diagnosis is critical and serves as the starting point for disease management. In this regard, Cas proteins play a significant role in managing diseases at the initial stages in plants. Cas proteins are attractive candidates for repurposing nucleic acid detection due to their programmability and extreme selectivity in binding and cleaving nucleic acids. Advances in understanding various Cas proteins have paved the way for the development of ultrasensitive, mobile, and cost-effective nucleic acid-based point-of-care (POC) testing equipment. Cas 9 proteins have been utilized to create robust and reliable nucleic acid detection technologies, but the recent discovery of Cas 13a and Cas 12a, with guaranteed cleavage activity, has revolutionized the field of nucleic acid detection (Gootenberg et al., 2017; Chen J. S. et al., 2018; Gootenberg et al., 2018; Azhar et al., 2021; Jiao et al., 2021).

**FIGURE 3:**
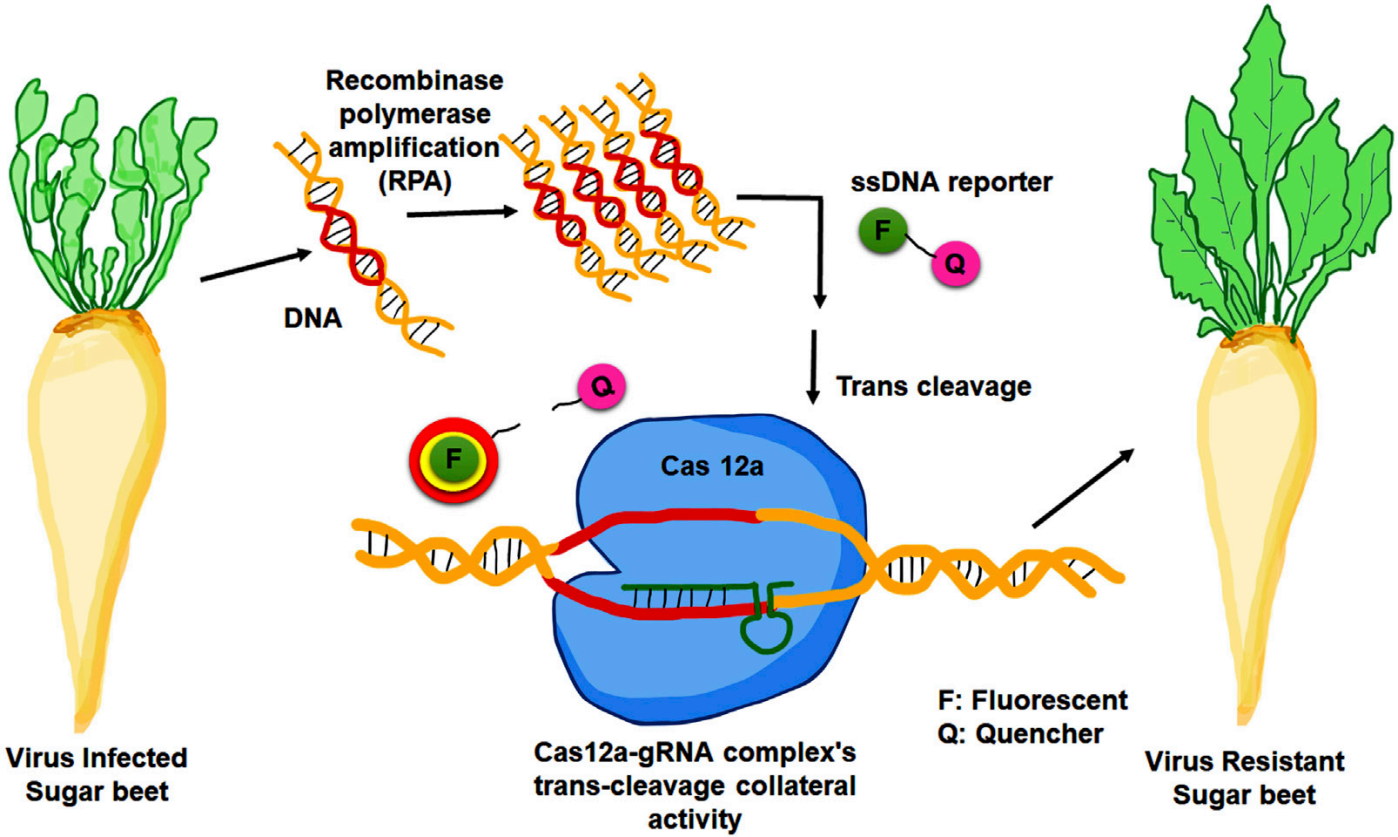
Development of viral resistance in sugar beet through DETECTR technology. The Cas12a-gRNA complex identifies target DNA that was amplified by recombinase polymerase amplification (RPA). When the target is recognised, it fragments the surrounding FQ-labeled ssDNA reporters, allowing the fluorescence to be recovered.

A new molecular diagnostic approach known as DETECTR (DNA Endonuclease Targeted CRISPR Trans Reporter) technology (Chen J. S. et al., 2018), based on the CRISPR-Cas 12a system had been utilized for detecting BNYVV in sugar beet roots (Ramachandran et al., 2021). In this diagnostic method, Cas 12a cleaves any surrounding single-stranded DNA without regard for its target. This trait is known as collateral activity, and it has been exploited to develop DETECTR. In this approach, a complementary guide RNA first directs Cas12a to a target dsDNA (Figure 3). When Cas 12a binds to the correct target, it cleaves ssDNA reporter molecules coupled with a quencher and a fluorophore. A fluorescence quencher (FQ)-labeled reporter was employed to monitor the trans-cleavage activity induced by the Cas12a-gRNA complex binding to the guide-complementary target DNA. In the presence of target DNA, the Cas 12a-gRNA complex’s trans-cleavage activity is triggered, leading to the cleavage of surrounding FQ-labeled ssDNA reporters (Chen J. S. et al., 2018). The fluorescent signal generated by the separation of the quencher and the fluorophore detects the indiscriminate cleavage (Kocak and Gersbach, 2018). DETECTR exhibits enhanced sensitivity when combined with RPA preamplification. This diagnostic technique requires the amplification of viral fragments from template DNA under isothermal conditions. The one-step reverse transcriptase recombinase polymerase amplification (RPA) method was employed in sugar beet. The precise sensitivity of this method is its standout feature, as it can detect a single molecule of viral particle within a microliter of the sample (Kocak and Gersbach, 2018).

Another common CRISPR-based technology applied in sugar beet for viral resistance is nucleic acid sequence-based amplification CRISPR cleavage (NASBA). This technology helped the plants achieve total viral resistance. This technique involves targeting viral DNA using a guide RNA (gRNA) and cutting the viral DNA with the Cas 9 enzyme. The trans-activating crRNA (tracrRNA) base pairs with the repeat sequence in the crRNA to form a unique dual RNA hybrid structure guide that directs Cas9 to cleave the target DNA. A chimeric sgRNA combines crRNA and tracrRNA into a single RNA transcript. The two nuclease domains (RuvC and HNH) present in Cas 9 cut the target and non-target DNA strands, respectively. A short trinucleotide PAM is also required for the initial target sequence identification; without it, the target sequence cannot be recognized. Successful identification results in a double-strand upstream of the 3′-NGG PAM (Li J. et al., 2022). This method is based on Cas 9 selective cleavage of target DNA and the toehold switch principle. This approach can distinguish between genotypes as it can identify a single base difference based on the presence or absence of a PAM sequence. This method utilizes sequence-based amplification, PAM-dependent target detection, Cas 9 cleavage, and a toehold sensor (Pardee et al., 2016).

**FIGURE 4:**
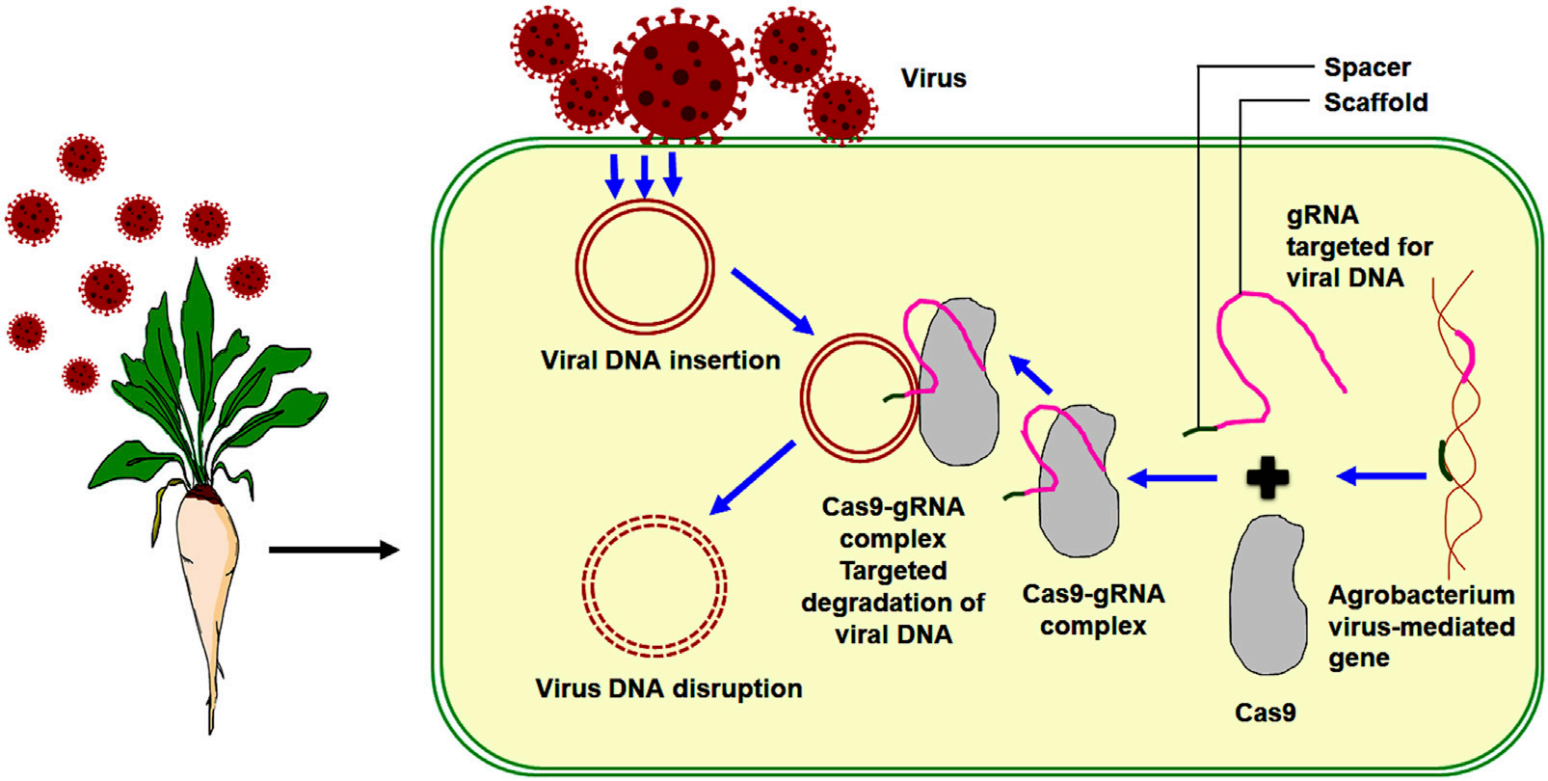
Viral resistance in sugar beet through CRISPR/Cas 9 system (Nucleic acid sequenced based amplification CRISPR cleavage).

Yildirim et al. (2019) utilized CRISPR/Cas 9 technology to confer various resistances in sugar beet against two curly top viruses (Beet curly top viruses (BCTV) and beet curly top Iranian viruses (BCTIV)). These viruses belong to two separate genera within the Geminiviridae family, specifically curtovirus and be-curtovirus families, respectively. The gRNA/cas-9 endonuclease system was transiently overexpressed to check BCTV and BCTIV in sugar beet plants. Sugar beet plants overexpressing the gRNA/Cas 9 constructs exhibited decreased viral DNA accumulation, and this accumulation was also observed to be delayed compared to plants without the overexpression of gRNA/Cas 9 constructs. The CRISPR/Cas 9 system used in providing resistance to sugar beet plants against viral diseases, specifically targets the dsDNA of a geminivirus with gRNA. This approach helps restrict virus reproduction by disrupting critical replication genes (Khatodia et al., 2017). Ji et al. (2015) demonstrated the application of CRISPR technology against geminivirus in plants. Efficient antivirus sgRNAs can be identified to recognize specific sites in the viral genome. This approach has high potential for developing multiple resistances to all Geminiviruses. The viral resistance mechanism in sugar beet through the CRISPR/Cas 9 system is illustrated in the figure below (Figure 4).

The advent of CRISPR/Cas 9 has opened up numerous prospects for developing superior crop varieties through genome editing with great precision and accuracy. CRISPR techniques usher in a new era of breeding systems in which plant immunity is enhanced by disrupting the compatible connection between infections and hosts (Karmakar et al., 2022). CRISPR-based available tools like SHERLOCK, FLASH, and LEOPARD wherein Cas 12 and Cas 13 proteins have been utilized, will be helpful in the identification, diagnosis and management of diseases in sugar beet.

### 4.3 Beet cyst nematode (*Heterodera schachtii* schmidt) resistance

The host range of the beet cyst nematode (BCN) is wide and includes numerous species from abundant plant families, including Chenopodiaceae and Brassicaceae. In sugar beet cultivation (*Beta vulgaris* L.), *Heterodera schachtii* is a serious pest. It is known that cultivated *Beta* species lack the genes that provide resistance to nematodes. *Beta procumbens*, a wild species, and its allied species (*B. webbiana* and *B. patellaris*) are the sources of resistance genes (Cai et al., 1997). BCN is resistant to the *Hs1* ^*pro*−1^ locus. The native *Hs1* ^*pro*−1^ gene encodes a 282-amino acid protein with incomplete leucine-rich repeats and a potential membrane-spanning region. This protein is produced in sugar beet roots. The expression of the matching complementary DNA gave resistance to BCN infection in susceptible sugar beet (Cai et al., 1997). The *Hs1* ^*pro*−1^ (Table 7) promoter promotes nematode feeding site-specific GUS expression in both sugar beet and *Arabidopsis*, indicating a shared mechanism for regulating *Hs1* ^*pro*−1^ expression in these two species (Thurau et al., 2003).

**TABLE 7:** CRISPR/Cas 9 technology application in major sugar beet diseases for pathogen (bacterial/fungal/viral) resistance.

Diseases	Causal organism	Potential genes imparting tolerance/resistance	Gene function	Yield loss (%)	References
Nematode associated diseases					
Beet cyst nematode	*Heterodera schachtii*	*Hs1^pro−1^*	Nematode resistance	Up to 60*	Cai et al. (1997)
		*Hs4*	Located on the wild beet translocation. Works together or independently of Hs1^pro−1^		Kumar et al. (2021); *Ghaemi et al. (2020)
Root-knot nematode	*Meloidogyne incognita*	*R6m-1*	Nematode resistance	Up to 50%	Bakooie et al. (2015)
		*Mi-sbp-1*	Regulator of lipogenesis		Shivakumara et al. (2019)
		*Mi-cpl-1*	Interaction between plants and nematodes		Dutta et al. (2015)
		*Mi-msp3*	Nematode resistance		Joshi et al. (2020)
		*Mi-msp5*			
		*Mi-msp18*			
		*Mi*-*msp24*			
		*Hs1* ^*pro−*^ *^1^*	General stress signalling genes and Nematode resistance		Abo-Ollo et al. (2018)
		*HSPRO2*			
		*Mi-1.2*	Nematode resistance		
		*R6m-1*			Bakooie et al. (2015)
Fungal diseases					
*Cercospora* leaf spot	*Cercospora beticola*	*SP1*	Acid chitinase activity	40*	Nielsen et al. (1994); Harvenson, (2013)
		*SP2*			Nielsen et al. (1994)
		*SE1*	Chitinase activity		Nielsen et al. (1993)
		*SE2*	Exochitinase activity		Nielsen et al. (1993)
		*qcr1*	(Qualitative Trait Loci) QTL disease resistance		Taguchi et al. (2011)
		*qcr4*			
*Rhizoctonia* root rot	*Rhizoctonia* sp.	*Rs1*	QTL disease resistance	50*	Lein et al. (2007); *Barry (2006)
		*Rs2*			
		*Rs3*			
*Fusarium* root rot	*F. oxysporum*	*BvSP2*	SNP markers	40–50	Yerzhebayeva. (2018)
		*BvSE2*	Chitinase activity		
Powdery mildew	*Erysiphe betae*	*Pm 1*	Partial resistance	Up to 35*	Francis and Luterbacher (2003); Lewellen and Schrandt (2001); Grimmer et al. (2007); *Neher and Gallian (2013); *Francis (2002)
		*Pm 2*			
		*Pm 3*	Complete resistance to disease		
		*Pm 4*	Partial resistance		
		*Pm 5*			
		*Pm 6*	Stronger resistance than others		
Aphanomyces seedling disease (Black rot or Black leg)	*Aphanomyces cochlioides*	*Acr 1*	Resistant gene	0–100	Taguchi et al. (2010); *Windels (2000)
Viral diseases					

Viral Diseases	Virus Family	Vector	Potential Genes Imparting tolerance/resistance	Yield loss (%)	References
Beet severe curly top virus (BSCTV)	Geminiviridae	Leafhopper	*Rep*	100*	*Agrios (2005); Strausbaugh et al. (2017)
			*Ty*		Gupta et al. (2021)
Beet necrotic yellow vein virus (BNYVV)	Benyvirus	*Polymyxa betae*	*Rz1 (the Holly gene)*	90*	Lewellen et al. (1987); Paul et al. (1993); Liebe et al. (2020)
			*Rz2*		Scholten et al. (1999); Wetzel et al. (2021)
			*Rz3*		Francis and Luterbacher (2003); *Pferdmenges (2007)

Kumar et al. (2021) identified the resistance gene, *Hs4* (about 230 kb-sized area). CRISPR-Cas-mediated deletion (CRISPRi) and overexpression (CRISPRa) in susceptible sugar beet roots were used to characterize a candidate gene. The gene encodes a rhomboid-like protease predicted to be bound to the endoplasmic reticulum. CRISPR Cas mutagenesis in the resistant sugar beet cultivar, NEMATA, deleted the ORF1. The roots of the knockout clones were extremely susceptible as huge numbers of J4 females and cysts packed with eggs observed in these roots. The expression of ORF in sugar beet roots determined nematode resistance/susceptibility. Beet roots expressing ORF 1 were resistant to nematodes while low ORF expression led to moderate susceptibility. Roots that did not express were susceptible to the nematode. Thus, CRISPRa can be a potential tool for the overexpressing targeted genes, which can confer tolerance to abiotic and biotic stress conditions (Horlbeck et al., 2016; Rai et al., 2019).

### 4.4 Beet necrotic yellow vein virus resistance

The multipartite genome of Beet necrotic yellow vein virus (BNYVV) consistis of five positive-stranded RNAs, providing an enticing framework for the production of several foreign proteins (Jiang et al., 2019). Additionally, *NbPDS* guide RNAs were delivered through BNYVV-based vectors to transgenic plants expressing Cas9 for genome editing. This delivery resulted in a photobleached phenotype in systemically infected leaves. The BNYVV-based vectors will facilitate the expression and production of multiple proteins in sugar beet and related crop plants. gRNA can be delivered using BNYVV-based vectors for CRISPR/Cas 9 plant genome editing (Jiang et al., 2019).

In a breeding line developed by the Holly Sugar Company in the USA, partial resistance to BNYVV was found to be caused by a single dominant gene (*Rz1*) (Lewellen et al., 1987; Scholten et al., 1996). Accessions WB42 (*Rz2*) and WB41 (*Rz3*) of *Beta vulgaris* subsp. maritima from Denmark have also been identified as having BNYVV resistance (Lewellen et al., 1987; Gidner et al., 2005). Compared to the *Rz1* gene, the dominant *Rz2* gene seems to confer a higher level of resistance (Paul et al., 1993).

Detection of BNYVV is extremely sensitive and specific to the infected roots, as measured by the reporter signal. Ramachandran et al. (2021) established isothermal RT-RPA and CRISPR-based virus diagnostic techniques for detecting BNYVV in sugar beet roots with rhizomanial disease. Using this CRISPR-based technique, BNYVV in sugar beet roots baited for rhizomania can be identified, generating a readily identifiable fluorescence signal as compared to healthy reference root samples. The BNYVV RNA-1 sequence was chosen as the target since it is one of the least diverged components of BNYVV. The CRISPR-Cas 12a-based BNYVV detection approach has paved the way for a sensitive, focused, and high-throughput detection platform for the assessment of rhizomania (Ramachandran et al., 2021).

The creation and validation of such CRISPR-based BNYVV diagnostic approaches for sugar beet roots offer advantages in terms of sensitivity and resilience in isothermal circumstances. Hence, it would be a helpful tool for the companies involved in evaluating viruses to drive disease control strategies (Ramachandran et al., 2021). Furthermore, the technology established for virus identification in underground root tissue can be applied to create a CRISPR-based detection platform for viruses and soil-borne disease-causing pathogens in other crops as well.

### 4.5 Beet curly top virus resistance

Beet curly top virus is a single stranded DNA virus that belongs to the genus Curtovirus and has been known to cause outbreaks in sugar beet as a disease. This virus has a strong impact on sugar beet yield, reducing it to 30% and above. The beet leaf hopper (*Circulifer tenellus*) is the vector of this disease (Strausbaugh et al., 2012). Yildirim et al. (2019) utilized 20 gRNAs which targeted viral DNAs of this virus, and the plant was transformed with Cas 9 enzyme including vector (pKIR1.1) to provide antiviral resistance. Viral movement is inhibited by the gRNA/Cas 9 construct. Overexpressing the gRNA/cas-9 constructs in sugar beet plants resulted in a delayed and diminished accumulation of both viral DNA. In this manner, sugar beet was harnessed to build full viral resistance in the species.

Yildrim et al. (2022) used a genome-based characterization technology of beet curly top Iranian virus in sugar beets and isolated and identified the Turkish variants of this virus along with its pathogenicity. Ebrahimi et al. (2022) explained the presence and pathogenicity of beet curly top Iranian virus in sugar beet. This helps in the management of this viral disease. Furthermore (Yildrim et al., 2023), reported that first-time broad-spectrum resistance against *Becurtovirus* using CRISPR technology where four gRNAs, involved in beet curly top Iranian virus, were cloned into vector consisting of Cas 9 and later agroinoculated into the virus infected leaves of sugar beet. Briddon et al. (1989) demonstrated that the genes for the capsid protein (CP), *C4*, and *C2* are essential for viral pathogenesis and the emergence of disease symptoms in plants. The CP gene mutation caused the virus to lose its infectiousness and ability to propagate. Targeting these genes through the CRISPR/Cas 9 tool in sugar beet will help in the management of this viral disease.

The importance of miRNAs in sugar beet curly top virus resistance (BCTV) has been shown. Majumdar et al. (2022) determined that differentially expressed (DE) miRNAs are, in certain cases, only present in the R lines. Future functional evaluation of these potential miRNAs, either by overexpression in germplasm that is BCTV sensitive or by employing them as molecular markers to scan various sugar beet genomes, will aid in establishing BCTV resistance. Furthermore, Eini et al. (2022) demonstrated the development of very effective geminiviral replicons (GVR) from BCTV with a broad host range for recombinant gene expression in plants.

Prior to the advent of molecular markers, only a single disease resistance gene (C gene) for BCTV resistance had been mapped in sugar beet (Mutasa-Gottgens et al., 2000). Studies have successfully developed markers linked to resistance genes for BNYVV (Luterbacher et al., 1998; Luterbacher et al., 2000), BCN (Owen and Ryser, 1942; Scholten et al., 1997) powdery mildew (Uphoff and Wricke, 1992) and to quantitative trait loci against *Cercospora* leaf spot.

### 4.6 *Cercospora* leaf spot resistance

In response to infection by the leaf-spot fungus *Cercospora beticola*, two isoforms of acid chitinase (*SE1* and *SE2*) were found in the leaves of sugar beet. However, only one of the isoforms (*SE2*) had exochitinase activity and could successfully hydrolyze chito-oligosaccharides (Nielsen et al., 1993). In resistant vs*.* susceptible cultivars, the sugar beet *SE2* gene (*B. vulgaris SE2*) is expressed at a substantially higher level following *Cercospora* infection (Nielsen et al., 1993). The leaves of sugar beet infected by *C. beticola* also contained two isoforms of another acid chitinase (*SP1* and *SP2*), which are similar to *SE1* and *SE2*. Infected sugar beet plant leaves had very high levels of *BvSP2* (*B. vulgaris* SP2) gene expression, however, *BvSP2* protein accumulation was only found in the vicinity of the infection sites (Nielsen et al., 1994).

Sugar beet leaves responded vigorously when exposed to a cell-wall protein solution made from the non-pathogenic oomycete *Pythium oligandrum* isolate. This response involved a substantial increase in *BvSE2* gene expression, which peaked at 4 h following the vaccination (Takenaka and Tamagake, 2009). However, since oomycetes lack chitin, *BvSE2* may be produced as part of a coordinated response to other proteins involved in the disease (Collinge et al., 1993).

### 4.7 Insect-pest resistance in sugar beet

Generally, resistance in insect-pest populations occur due to mutations in genes, which facilitates the encoding for receptor molecules and disrupt the interaction between the insect and the toxin. In rice, CRISPR/Cas9-dependent knockout of *CYP71A1* mutant gene enables it encode a functional tryptamine 5- hydroxylase (Lu et al., 2018). Tryptamine 5-hydroxylase is responsible for transforming tryptamine to serotonin and increases plant resistance against plant hoppers. A similar approach could be applied to the sugar beet plants to develop resistance against leaf chewing insects like *H. armigera* and *S. litura*. CRISPR/Cas 9-mediated mutagenesis of *GmUGT* led to the development of transgenic plant (Zhang et al., 2022). The *Arabidopsis ugt72b1* mutant exhibited aggravated cell wall lignification and an increase in flavonoid content (Lin et al., 2016). Both cell wall lignification and flavonoids contribute to resistance against leaf-chewing insects. Cell wall lignification serves as the first physical barrier against leaf-chewing insects (War et al., 2012). Hence, the enhanced resistance against leaf-chewing insects due to GmUGT mutations could be attributed to aggravated cell wall lignification and altered flavonoids (Zhang et al., 2022). Sugar beet plants transformed with the *cry1Ab* gene exhibited powerful defense against lepidopteron insects (Jafari et al., 2009). Sedighi et al. (2011) also reported a similar success story against an Egyptian leafworm (*Spodoptera littoralis*) infestation. Sugar beet plants have been transformed with *Cry1Ab* and *Cry1C* which exhibit resistance against the cabbage armyworm (*Spodoptera frugiperda*) (Kimoto and Shimamoto, 2001; Kimoto and Shimamoto, 2002) and on lepidopteron with *Cry1C* and *Cry2A* as well (Lytvyn et al., 2014). Regev et al. (1996) described that the *Cry1C* protein controls larvae of *Spodoptera* spp.

One of the most efficient methods for pest control is utilizing the toxic qualities of Cry protein’s present in the Gram-positive pathogenic bacterium *Bacillus thuringiensis*. Different families of *Cry* proteins (or *Bt* proteins) exhibit highly selective toxicity against members of specific insect orders (Lytvyn et al., 2014). The order Lepidoptera exhibits a toxic response against proteins encoded by *Cry1* and *Cry9* genes (Table 8). Diptera and Lepidoptera show toxicity against proteins encoded by *Cry2* genes. Coleopteran insects exhibit a toxic response against proteins encoded by *Cry3* genes. Diptera also show a toxic response against the protein encoded by the *Cry2* gene (Yuan et al., 2007).

**TABLE 8:** Genes that can impart tolerance to sugar beet crop against insect pests (larval stage) *via* CRISPR/Cas 9 technology.

(a) Some genomes successfully edited in insects by the CRISPR/Cas tool kits						
Order	Insect name (common name)	Gene	Gene function	Editing	Outcome of the editing	References
		Imparting tolerance/resistance				
Lepidoptera	*Spodoptera litura* Fabricius (Armyworm)	*Slit PBP3*	Sex pheromone perception	Chimera mutation	Destroyed pest insect mating	Zhu et al. (2016)
		*siSe2* (Serine protease 2)	Sperm movement and activity	Knockout	Induces male sterility	Bi et al. (2022)
		*Abdominal-A (slabd-A)*	• Embryonic development gene	Knockout	Defected body segmentation and irregular pigmentation	Bi et al. (2016); Sun et al. (2017)
			• Body segmentation			
		*SlitBLOS2*	Acts as a marker gene	Knockout	Coloration of the integuments, a marker gene for functional studies and pest control strategies	Zhu et al. (2017)
	*Spodoptera frugiperda* J.E. Smith (Fall armyworm)	*BLOS2* (Biogenesis of lysosome-related organelles complex 1 subunit 2)		Knockout	Translucent mosaic integument	Zhu et al. (2020)
		*TO* (Tryptophan 2, 3-dioxygenase)			Olive eye color	
		*E93*	A key ecdysone-induced transcription factor that promotes adult development		Larval-pupal intermediate phenotypes	
		*ABCB1*	Susceptibility to chemical pesticides or Bt toxins	Knockout	Susceptibility to emamectinbenzoate, beta-cypermethrin and chlorantraniliprole	Li et al. (2022b)
	*Spodoptera exigua* (Beet armyworm)	*CYP9A186*	Restoration of Emamectin benzoate (EB) [(4″R)-4″-deoxy-4” -(methylamino) avermectin B1 benzoate] susceptibility	Knockout	Susceptibility to emamectin benzoate (EB)	Zuo et al. (2021)
		Ryanodine receptor	Regulates calcium release from intracellular stores and other cellular processes, *viz.,* muscle contraction, gene transcription, neurotransmitter release, hormone secretion and cell proliferation	Substitution	Controlled insect population and resistance to various insecticides	Zuo et al. (2017)
		P-glycoprotein gene	Unknown	Knockout	Susceptibility to abamectin and emamectin benzoate	Jin et al. (2020)
		Seα6 (*Spodoptera exigua* a-6-nicotinic acetylcholine receptor (nAchR))	Role in Spinosyns insecticide interaction	Knockout	Resistance to spinosyn insecticides	Zuo et al. (2018); Zuo et al. (2020)
	*Spodoptera littoralis* (Egyptian cotton leafworm)	Orco (Odorant receptor co-receptor)	Impairs feeding, mating and egg-laying behavior	Knockout	Reduced survival rate	Koutroumpa et al. (2016)
		SlitOrco	plant odor and sex pheromone olfactory detection	Knockout	Investigated the function of the *Orco*	Cui et al. (2017); Koutroumpa et al. (2016)
					gene in the non-model insect	
					*Spodoptera littoralis*	
	*Agrotis ipsilon* Rott. (Cutworm)	*Yellow-y*	Involved in body pigmentation and play a role in waterproofing	Knockout	Pigmentation plays a vital role in insect survival and reproduction	Chen et al. (2018a)
		*AiTH* (*Agrotis ipsilon* tyrosine hydroxylase)	Insect melanin and catecholamine biosynthesis pathway	Knockout	• Narrowing in the eggshell	Yang et al. (2018)
					• Pigmentation of epidermis and newly hatched larval development	
		*Aidsx*	Embryonic development	Knockout/Disruption	Sexually dimorphic development and behavior	Chen et al. (2019)
		*AiMasc* (*Masculinizer gene*)	Sex determination		Induced expression of male specific double sex isoform	Wang et al. (2019)
	*Helicoverpa armigera* Hübner (Gram pod borer)	*GmUGT* (QTL-M)	Alteration of flavonoid biosynthesis pathway	Insertion	Resistance to insect	Zhang et al. (2022)
		HaCad (*Helicoverpa armigera* cadherin)	As receptor for Bt Cry 1A Toxin	Knockout	Resistance to Bt toxin Cry1Ac	Wang et al. (2016)
		Cluster of nine P450 genes	Defense mechanism against natural/synthetic insect toxins	Knockout	Identification of the key players in the insecticide metabolism	Wang et al. (2018)
		*CYP6AE*		Knockout	Regulation of detoxification enzymes	Wang et al. (2018)
		*OR16*	Pheromone antagonist	Knockout	Destroyed pest insect mating	Chang et al. (2017)
		*Tetraspanin*	Cell migration, signal transduction, and intracellular trafficking	Knockout	Resistance to Bt toxin cry1Ac	Jin et al. (2018)
		*HaABCA2*	Involved in the resistance mechanism for Cry2Ab	Knockout	Resistance to cry2Aa and cry2Ab	Wang et al. (2017a)
		*White*	Differential distribution of eye pigments	Knockout	Patterns of pigmentation	Khan et al. (2017)
		*Brown*			No phenotypic effects on viability or pigmentation	
		*Scarlet*			Increase amount of pteridines or ommochromes	
		*ok*	Analogous to that of brown gene in *Drosophila*			
		*NPC1b*	Growth of *Helicoverpa armigera* larvae and dietary cholesterol uptake	Knockout	Upregulation in gene expression during early larval instars	Zheng et al. (2020)

(b) Some genomes successfully edited in plants by the CRISPR/Cas tool kits against insects							
Order	Insect Name (Common Name)	Gene	Crop reported in	Gene Function	Editing	Outcome of the editing	References
		Imparting tolerance/resistance					
Lepidoptera	*Spodoptera frugiperda* J.E. Smith (Fall armyworm)	*Cry1Fa*	Field crop	Practically resistance	Knockout	SeABCC2 has a major role and SeCad1 a minor role in mediating toxicity of Cry1Ac and Cry1Fa	Huanga et al. (2020); Flagel et al. (2018); Jin et al. (2020)
		*SfABCC2*		Resistance to *Cry1F* but no alteration in susceptibility to small molecule pesticides	Knockout	Cry1F due to mutations in the SfABCC2 gene do not affect susceptibility to the synthetic and semisynthetic small molecule pesticides	Abdelgaffar et al. (2020); Jin et al. (2021)
		*Cry1Ac*	Field crop	(SfCad) cadherin protein	Knockout mutant strain	Targets different exons of the *SfCad* gene	Zhang et al. (2020a)
		Cry1Ab		Toxin protein			Zhang et al. (2020a); Kimoto and Shimamoto (2001); Hernández-Rodríguez et al. (2013); Jin et al. (2020)
		*Cry1C*		(SfCad) cadherin protein			
		*ABC transporters*		As receptor for Bt Cry1Fa and Cry1Ab toxins	Knock out	• Toxicity of two *bacillus* thuringiensis cry1 toxins to the pest	Jin et al. (2020)
						Resistance to both cry1fa and cry1ab toxins	
	*Spodoptera littoralis* (Egyptian cotton leafworm)	*Cry1Ab*	Sugar beet	Toxin protein	Insertion	• Strong anti-feedant effect on insect	Sedighi et al. (2011)
						• Increase in the developmental time and mortality	
	*Helicoverpa armigera* Hübner (Gram pod borer)	*Cry1Ac*	Field crop	Cadherin as a receptor, Toxin protein	Insertion	Saturable, high-affinity binding to insect	Stewart et al. (2001); Wang et al. (2005); Wang et al. (2016); Chen et al. (2018b); Karim et al. (2000)
		*Cry2Ab*			Knockout	High levels of resistance	Chen et al. (2018b); Wang et al. (2017a)
		*GmUGT* (QTL-M)	Soyabean	Alteration of flavonoid biosynthesis pathway	Insertion	Overexpression of GmUGT produced sensitive soybean varieties against *H. armigera*	Zhang et al. (2022)
		Cry2Aa	Pigeon pea	Mediate toxicity	Knockout	High levels of resistance	Wang et al. (2017a); Singh et al. (2018)
Diptera	All species	*Cry2*	Multiple crops	Toxin protein	• Potential gene that can be targeted for CRISPR/Cas 9		Yuan et al. (2007)
					• Transcription repression causes lack in ability to repress Clock: cycle mediated transcription		
	*Tetanops myopaeformis* (Sugar beet root maggot)	Delta-endotoxin genes	Sugar beet	Target an insect’s digestive system	Insertion	Resistance	Wilhite et al. (2000); Smigocki et al. (2003)
Coleopteran	All species	*Cry3*	Multiple crops	Toxin protein	• Potential gene that can be targeted for insecticidal *Bt* toxins using CRISPR/Cas 9		Orduz et al. (1996); Pauchet et al. (2016)

## 5 Conclusion and future prospects

Global laboratories are increasingly turning to CRISPR/Cas 9 editing as their instrument of choice for determining how genes work and how they might be used in other contexts. This technology is being utilized in various crop development efforts to reduce biotic and abiotic stressors. The great precision, efficacy, efficiency, cost-effectiveness, and time efficiency of editing procedures have led to their development as useful tools. The advancement in molecular tools like CRISPR/Cas 9 has opened up new approaches for genome editing in sugar beet. This technology can be used for the generation of resistant/tolerant sugar beet breeding lines/germplasm to withstand abiotic/biotic stress. In sugar beet, specific genes can be silenced or knocked out to change their functionality. The plant may benefit and adapt to the abiotic stress environment. Tolerance to such circumstances may be linked to adjustments in their physiological and biochemical mechanisms. The subsequent breeding cycles produces sugar beet cultivars that are more resilient to such challenges due to the adoption of carefully chosen tolerant breeding lines. Under drought stress conditions, this improvement becomes apparent in the plant, highlighting the plant’s enhanced water usage efficiency. This can also be tested for sugar beet. Furthermore, investigations into the use of CRISPR/Cas 9 to create novel quantitative features/traits with gain-of-function mutations through replacements in sugar beet could be seen. CRISPR/Cas 9 technology promises to make a significant contribution to understanding the gene regulatory networks underlying abiotic stress response/adaptation and crop improvement initiatives to create stress-tolerant plants.

Additionally, CRISPR/Cas 9 technology applications hold great potential for addressing the challenges faced in sugar beet crops during biotic stress. Biotic stresses, resulting from pathogen and pest occurrences, significantly affect sugar beet production and yield. Traditional breeding methods have certain limitations in achieving rapid and precise genetic modification. CRISPR/Cas 9 technology has emerged as a promising solution with revolutionary approach to improve sugar beet tolerance to biotic stresses. Despite success of CRISPR/Cas 9 technology in controlling biotic stress, particularly diseases in economically significant crops, its use in insect management has not been fully utilized. Modest success has been achieved despite the intellectual exercise in creating techniques for insect pest resistance in both insects and plants. In contrast to other stresses, the main drawback has been the scarcity of target genes. Therefore, it is crucial for scientists to focus on finding sources of resistance that might serve as a foundation for insect control. To achieve this, it is necessary to evaluate the available germplasm, including wild relatives of certain crops, for pest response and to identify stress-responsive genes using multi-omics techniques. Targeted mutations turning susceptible plants into those that can control their respective pests are not far off in space or time, with such studies already in vogue. These factors, along with regulatory restrictions on gene-edited crops, may help the technique succeed in advancing not only science but also societal acceptance.

## Figures and Tables

**Table:** 

**aaNAT**	Arylalkylamine-N-acetyltransferase
**Ag**	Silver
**APX**	Ascorbate Peroxidase
**As**	Arsenic
**BBMV**	Brush-border membrane vesicles
**BCTV**	Beet Curly Top Virus Resistance
**BNYVV**	Beet Necrotic Yellow Vein Virus
**CAT**	Catalase
**Cd**	Cadmium
**Co**	Cobalt
**CP**	Capsid Protein
**Cr**	Chromium
**CRISPR/Cas**	Clustered Regularly Interspaced Short Palindromic Repeats
**CRISPRa**	CRISPR Activation
**CRISPRi**	CRISPR Interference
**crRNA**	CRISPR RNA
**CRY**	Crystalline Protein
**DE**	Differentially Expressed
**DETECTR**	DNA Endonuclease Targeted CRISPR Trans Reporter
**dsRNA**	Double-Stranded RNA
**Fe**	Iron
**FQ**	Fluorescence Quencher
**GR**	Glutathione Reductase
**GVR**	Geminiviral Replicons
**HD-RNAi**	Host-Delivered RNA Interference
**HIGS**	Host-Induced Gene Silencing
**HSFs**	Heat Shock Transcription Factors
**HSPs**	Heat Shock Proteins
**HSEs**	Heat Shock Elements
**ICPs**	Insecticidal Crystal Proteins
**KRAB**	Kruppel Associated Box
**lncRNAs**	Long noncoding RNAs
**NADA**	*N-*acetyl dopamine
**Ni**	Nickel
**NIC**	Toxic Nickel Concentration
**PAM**	Protospacer Adjacent Motif
**Pb**	Lead
**PI**	Protease Inhibitor
**PM**	Peritrophic Membrane
**POC**	Point-of-Care
**Pt**	Platinum
**RKN**	Root-Knot Nematode
**ROS**	Reactive oxygen species
**RPA**	Recombinase Polymerase Amplification
**SBRM**	Sugar beet root maggot
**sgRNA**	Short Guide RNA
**SOD**	Superoxide Dismutase
**TALENs**	Transcription Activator Like Effector Nucleases
**TSS**	Transcription Start Site
**VIP**	Vegetal Insecticidal Protein
**ZFNs**	Zinc-Finger Nucleases
**Zn**	Zinc

## References

[B1] Abdelgaffar H. Perera O. P. Jurat-Fuentes J. L. (2020). ABC transporter mutations in Cry1F-resistant fall armyworm (Spodoptera frugiperda) do not result in altered susceptibility to selected small molecule pesticides. Pest Manag. Sci. 77 (2), 949–955. 10.1002/ps.6106 32985759

[B2] Abo-Ollo N. A. Abdel-Rahman M. M. Saleh M. S. Gohar I. M. A. (2018). Differentiate between sugar beet (*Beta vulgaris* L) genotypes resistance to root knot nematode (*Meloidogyne incognita*) by molecular markers. J. Agri. Chem. Biotec. Mansoura Univ. 9 (8), 189–194. 10.21608/jacb.2018.35234

[B3] Abou-Elwafa S. F. Amin A. E. A. Eujayl I. (2020). Genetic diversity of sugar beet under heat stress and deficit irrigation. Agron. J. 112, 3579–3590. 10.1002/agj2.20356

[B4] Aglawe S. N. Barbadikar K. M. Mangrauthia S. K. Madhav M. S. (2018). New breeding technique genome editing for crop improvement: applications, potentials and challenges. 3 Biotech 8 (8), 336. 10.1007/s13205-018-1355-3 PMC6056351 30073121

[B5] AgResearch Magazine (2016). New sugar beet persist curly top virus. US department of Agriculture. https://agresearchmag.ars.usda.gov/2016/mar/sugarbeet/.

[B6] Agrios G. N. (2005). “Plant diseases caused by nematodes,” in Plant Pathology Editor Agrios G. N. (Burlington, MA, USA: Elsevier Academic Press) 825–874. 10.1016/B978-0-08-047378-9.50021-X

[B7] Ahmad A. Khan S. H. Khan Z. (2021). CRISPR crops: the future of food security. Springer Nature, Singapore Pte Ltd, 289. 10.1007/978-981-15-7142-8

[B8] Akram F. Sahreen S. Aamir F. Haq I. U. Malik K. Imtiaz M. (2023). An insight into modern targeted genome editing technologies with a special focus on CRISPR/Cas9 and its applications. Mole. Biotech. 65, 227–242. 10.1007/s12033-022-00501-4 PMC9041284 35474409

[B9] Alfatih A. Wu J. Jan S. U. Zhang Z. S. Xia J. Q. Xiang C. B. (2020). Loss of rice PARAQUAT TOLERANCE 3 confers enhanced resistance to abiotic stresses and increases grain yield in field. Plant Cell Environ. 43 (11), 2743–2754. 10.1111/pce.13856 32691446

[B10] Anagholi A. Rajabi A. Khayamim S. (2018). Response of sugar beet genotypes under salinity stress in Central areas of Iran. Int. J. Pharm. Phytopharm. Res. 8 (6), 49–58. https://eijppr.com/r8Be2ZB.

[B11] Asmamaw M. Zawdie B. (2021). Mechanism and applications of CRISPR/Cas-9-mediated genome editing. Biologics 15, 353–361. 10.2147/BTT.S326422 34456559 PMC8388126

[B12] Azhar M. Phutela R. Kumar M. Ansari A. H. Rauthan R. Gulati S. (2021). Rapid and accurate nucleobase detection using FnCas9 and its application in COVID-19 diagnosis. Biosens. Bioelectron. 183, 113207. 10.1016/j.bios.2021.113207 33866136 PMC8020606

[B13] Badhan S. Ball A. S. Mantri N. (2021). First report of CRISPR/Cas9 mediated DNA-free editing of 4CL and RVE7 genes in Chickpea protoplasts. Int. J. Mol. Sci. 22, 396. 10.3390/ijms22010396 33401455 PMC7795094

[B14] Baeg G. J. Kim S. H. Choi D. M. Tripathi S. Han Y.-J. Kim J.-I. (2021). CRISPR/Cas9-mediated mutation of 5-oxoprolinase gene confers resistance to sulfonamide compounds in *Arabidopsis* . Plant Biotechnol. Rep. 15 (6), 753–764. 10.1007/s11816-021-00718-w

[B301] Baitha A. Srivastava S. Misra V. (2022). “Insect-pests of sugar beet and their integrated management,” in Sugar Beet Cultivation, Management and Processing. Editors Misra V. Srivastava S. Mall A. K. (Singapore: Springer), 643–657. 10.1007/978-981-19-2730-0_31

[B15] Bajpai A. B. Solanki A. Srivastava N. Semwal P. P. Thapliyal P. Payal R. (2023). CRISPR-Cas System: a revolutionizing tool for genome editing. Biochem. *Cell. Arch*. 23 (1), 623–630. 10.51470/bca.2023.23.1.623

[B16] Bakooie M. Pourjam E. Mahmoudi S. B. Safaie N. Naderpour M. (2015). Development of an SNP marker for sugar beet resistance/susceptible genotyping to root-knot nematode. J. Agr. Sci. Tech. 17, 443–454.

[B17] Barakate A. Stephens J. (2016). An overview of CRISPR-based tools and their improvements: new opportunities in understanding plant-pathogen interactions for better crop protection. Front. Plant Sci. 7, 765. 10.3389/fpls.2016.00765 27313592 PMC4887484

[B18] Barratt G. E. Murchie E. H. Sparkes D. L. (2023). Water use efficiency responses to fluctuating soil water availability in contrasting commercial sugar beet varieties. Front. Plant Sci. 14, 1119321. 10.3389/fpls.2023.1119321 36968376 PMC10034331

[B19] Barry J. J. (2006). Root rot disease of sugar beet. Zb. Matice Srp. za Prir. nauke, 110. 10.2298/ZMSPN0610009J

[B20] Beata P. Roszival M. Kubova V. (2022). Influence of heavy metals on growth and metabolism of sugar beet. Lsity Cukrovanicke a Reparske 138 (3), 112–115.

[B21] Bhatia S. Pooja Yadav S. K. (2023). CRISPR-Cas for genome editing: classification, mechanism, designing and applications. Int. J. Biol. Macromol. 238, 124054. 10.1016/j.ijbiomac.2023.124054 36933595

[B22] Bhattacharya A. (2022). “Plant growth hormones in plants under low-temperature stress: a review,” in Physiological processes in plants under low temperature stress. Editor Bhattacharya A. (Singapore: Springer), 517–627. 10.1007/978-981-16-9037-2_6

[B23] Bi H. Xu X. Li X. Wang Y. Zhou S. Huang Y. (2022). CRISPR/Cas9-mediated *Serine protease 2* disruption induces male sterility in *Spodoptera litura* . Front. Physiol. 13, 931824. 10.3389/fphys.2022.931824 35991171 PMC9382020

[B24] Bi H. L. Xu J. Tan A. J. Huang Y. P. (2016). CRISPR/Cas9-mediated targeted gene mutagenesis in *Spodoptera litura* . Insect Sci. 23, 469–477. 10.1111/1744-7917.12341 27061764

[B25] Bolotin A. Quinquis B. Sorokin A. Ehrlich S. D. (2005). Clustered regularly interspaced short palindrome repeats (CRISPRs) have spacers of extrachromosomal origin. Microbiologia 151 (8), 2551–2561. 10.1099/mic.0.28048-0 16079334

[B26] Boti M. A. Athanasopoulou K. Adamopoulos P. G. Sideris D. C. Scorilas A. (2023). Recent advances in genome engineering strategies. Genes 14 (1), 129. 10.3390/genes14010129 36672870 PMC9859587

[B27] Bozdag G. O. Kaya A. Koc A. Noll G. A. Prüfer D. Karakaya H. C. (2014). Characterization of a cDNA from *Beta maritima* that confers nickel tolerance in yeast. Gene 538, 251–257. 10.1016/j.gene.2014.01.052 24487090

[B28] Bravo A. Gill S. S. Soberón M. (2007). Mode of action of *Bacillus thuringiensis* Cryand Cyt toxins and their potential for insect control. Toxicon 49 (4), 423–435. 10.1016/j.toxicon.2006.11.022 17198720 PMC1857359

[B29] Briddon R. W. Watts J. Markham P. G. Stanley J. (1989). The coat protein of beet curly top virus is essential for infectivity. Virol. 2, 628–633. 10.1016/0042-6822(89)90205-5 2800340

[B30] Buchholzer M. Frommer W. B. (2023). An increasing number of countries regulate genome editing in crops. New Phytol. 237, 12–15. 10.1111/nph.18333 35739630

[B31] Butt H. Rao G. S. Sedeek K. Aman R. Kamel R. Mahfouz M. (2018). Engineering herbicide resistance *via* prime editing in rice. Plant Biotechnol. J. 18 (12), 2370–2372. 10.1111/pbi.13399 PMC7680537 32415890

[B32] Bybordi A. (2010). Effects of salinity on yield and component characters in canola (*Brassica napus* L.) cultivars. Not. Sci. Biol. 2 (1), 81–83. 10.15835/nsb.2.1.3560

[B33] Cai D. Kleine M. Kifle S. Harloff H. J. Sandal N. N. Marcker K. A. (1997). Positional cloning of a gene for nematode resistance in sugar beet. Sci 275, 832–834. 10.1126/science.275.5301.832 9012350

[B34] Carte J. Wang R. Li H. Terns R. M. Serns M. P. (2008). Cas6 is an endoribonuclease that generates guide RNAs for invader defense in prokaryotes. Genes Dev. 22 (24), 3489–3496. 10.1101/gad.1742908 19141480 PMC2607076

[B35] Casarini B. (1999). “Le annersità: loro natura, prevenzione e lotta,” in La barbabietola negli ambienti mediterranei. Editors Casarini B. Biancardi E. Ranalli P. (Bologna, Italy: Edagricole), 273–421.

[B36] Chang H. Liu Y. Ai D. Jiang X. Dong S. Wang G. (2017). A pheromone antagonist regulates optimal mating time in the moth *Helicoverpa armigera* . Curr. Biol. 27, 1610–1615. 10.1016/j.cub.2017.04.035 28528905

[B37] Chang J. D. Huang S. Yamaji N. Zhang W. Ma J. F. Zhao F. J. (2020). OsNRAMP1 transporter contributes to cadmium and manganese uptake in rice. Plant Cell Environ. 43, 2476–2491. 10.1111/pce.13843 32666540

[B38] Chaturvedi S. (2004). Biosafety regulation: need for fine balancing. Econ. Polit. Wkly. 39, 3693–3697.

[B39] Chaudhuri A. Halder K. Datta A. (2022). Classification of CRISPR/Cas system and its application in tomato breeding. Theor. Appl. Genet. 135, 367–387. 10.1007/s00122-021-03984-y 34973111 PMC8866350

[B40] Chen J. S. Ma E. Harrington L. B. Costa M. D. Tian X. Palefsky M. (2018a). CRISPR-Cas12a target binding unleashes indiscriminate single stranded DNase activity. Sci 360 (6387), 436–439. 10.1126/science.aar6245 PMC6628903 29449511

[B41] Chen K. Gao C. (2020). Genome edited crops: how to move them from laboratory to market. Front. Agr. Sci. Eng. 7 (2), 181–187. 10.15302/J-FASE-2020332

[B42] Chen L. Wei J. Liu C. Zhang W. Wang B. Niu L. (2018b). Specific binding protein ABCC1 is associated with Cry2Ab toxicity in *Helicoverpa armigera* . Front. Physiol. 19 (9), 745. 10.3389/fphys.2018.00745 PMC6018205 29971014

[B43] Chen X. Cao Y. Zhan S. Tan A. Palli S. R. Huang Y. (2019). Disruption of sex-specific double sex exons results in male-and female-specific defects in the black cutworm, *Agrotis ipsilon* . Pest Manag. Sci. 75 (6), 1697–1706. 10.1002/ps.5290 30520231

[B44] Chen X. Cao Y. Zhan S. Zhang Y. Tan A. Huang Y. (2018c). Identification of yellow gene family in *Agrotis ipsilon* and functional analysis of Aiyellow-y by CRISPR/Cas 9. Insect biochem. Mol. Biol. 94, 1–9. 10.1016/j.ibmb.2018.01.002 29337139

[B45] Chinnusamy V. Zhu J. Zhu J. K. (2006). Gene regulation during cold acclimation in plants. Physiol. Plant. 126 (1), 52–61. 10.1111/j.1399-3054.2006.00596.x

[B46] Chu C. Huang R. Liu L. Tang G. Xiao J. Yoo H. (2022). The rice heavy-metal transporter OsNRAMP1 regulates disease resistance by modulating ROS homoeostasis. Plant Cell Environ. 45, 1109–1126. 10.1111/pce.14263 35040151

[B47] Collinge D. B. Kragh K. M. Mikkelsen J. D. Nielsen K. K. Rasmussen U. Vad K. (1993). Plant chitinases. Plant J. 3 (1), 31–40. 10.1046/j.1365-313x.1993.t01-1-00999.x 8401605

[B48] Cooke D. A. (1987). Beet cyst nematode (Heterodera Schachtii Schmidt) and its control on sugar beet. Agricul. Zool. Rev. 2, 135–183.

[B49] Cui Y. Sun J. L. Yu L. (2017). Application of the CRISPR gene-editing technique in insect functional genome studies - a review. Entomol. Exp. Appl. 162 (2), 124–132. 10.1111/eea.12530

[B50] Davis C. A. Hitz B. C. Sloan C. A. Chan E. T. Davidson J. M. Gabdank I. (2018). The encyclopedia of DNA elements (ENCODE): data portal update. Nucleic Acids Res. 46 (1), D794–D801. 10.1093/nar/gkx1081 29126249 PMC5753278

[B51] Deveau H. Barrangou R. Garneau J. E. Labonté J. Fremaux C. Boyaval P. (2008). Phage response to CRISPR-encoded resistance in Streptococcus thermophilus. J. Bacteriol. 190 (4), 1390–1400. 10.1128/JB.01412-07 18065545 PMC2238228

[B52] DiFonzo C. Jewett M. Warner F. Brown-Rytlewski D. Kirk W. (2006). Insect, nematode, and disease control in Michigan field crops. MSU Bulletin E-1582 2006. Michigan State University East Lansing, MI 48824 https://www.canr.msu.edu/field_crops/uploads/archive/Part18E1582DryBeanDiseases.pdf.

[B53] Dobrovidova O. (2019). Russia joins in global gene-editing bonanza. Nature 569, 319–320. 10.1038/d41586-019-01519-6 31089233

[B54] Du Y. T. Zhao M. J. Wang C. T. Gao Y. Wang Y. X. Liu Y. W. (2018). Identification and characterization of GmMYB118 responses to drought and salt stress. BMC Plant Biol. 18, 320. 10.1186/s12870-018-1551-7 30509166 PMC6276260

[B55] Duan Y. B. Li J. Qin R. Y. Xu R. F. Li H. Yang Y. C. (2016). Identification of a regulatory element responsible for salt induction of rice OsRAV2 through ex-situ and *in situ* promoter analysis. Plant Mol. Biol. 90, 49–62. 10.1007/s11103-015-0393-z 26482477

[B56] Dutta T. K. Papolu P. K. Banakar P. Chiudhary D. Sirohi A. Rao U. (2015). Tomato transgenic plants expressing hairpin construct of a nematode protease gene conferred enhanced resistance to root knot nematodes. Front. Microbiol. 6, 260. 10.3389/fmicb.2015.00260 25883594 PMC4381642

[B57] Ebrahimi S. Alexandra Babler A. Eini O. Yildirim Z. Wassenegger W. Krczal G. (2022). Beet curly top Iran virus Rep and V2 gene work as silencing suppressors through separate mechanisms. bioRxiv. 10.1101/2022.08.25.505242

[B58] Eini O. Schumann N. Niessen M. Varrelmann M. (2022). Targeted mutagenesis in plants using Beet curly top virus for efficient delivery of CRISPR/Cas12a components. New Biotech. 67, 1–11. 10.1016/j.nbt.2021.12.002 34896246

[B59] Erbasol I. Bozdag G. O. Koc A. Pedas P. Karakaya H. C. (2013). Characterization of two genes encoding metal tolerance proteins from *Beta vulgaris* subspecies maritima that confers manganese tolerance in yeast. BioMetals 26, 795–804. 10.1007/s10534-013-9658-7 23864431

[B60] Esh A. Taghian S. (2022). “Etiology, epidemiology, and management of sugar beet diseases,” in Sugar beet cultivation, management and processing. Editors Misra V. Srivastava S. Mall A. K. (Singapore: Springer), 505–540. 10.1007/978-981-19-2730-0_25

[B61] Fan K. Mao Z. Ye F. Pan X. F. Li Z. Lin W. (2021). Genome-wide identification and molecular evolution analysis of the heat shock transcription factor (HSF) gene family in four diploid and two allopolyploid *Gossypium* species. Genomics 113 (5), 3112–3127. 10.1016/j.ygeno.2021.07.008 34246694

[B62] Fang S. Hou X. Liang X. (2021). Response mechanisms of plants under saline-alkali stress. Front. Plant Sci. 12, 667458. 10.3389/fpls.2021.667458 34149764 PMC8213028

[B63] Fang Y. Xiong L. (2015). General mechanisms of drought response and their application in drought resistance improvement in plants. Cell Mol. Life Sci. 72 (4), 673–689. 10.1007/s00018-014-1767-0 25336153 PMC11113132

[B64] FAO (2022). Agriculture statistics. Rome, Italy: Food and Agriculture Organization of the United Nations.

[B65] Fineran P. C. Gerritzen M. J. Suárez-Diez M. Künne T. Boekhorst J. van Hijum S. A. (2014). Degenerate target sites mediate rapid primed CRISPR adaptation. PNAS 111 (16), E1629–E1638. 10.1073/pnas.1400071111 24711427 PMC4000823

[B66] Flagel L. Lee Y. W. Wanjugi H. Swarup S. Brown A. Wang J. (2018). Mutational disruption of the ABCC2 gene in fall armyworm *Spodoptera frugiperda* confers resistance to the Cry1Fa and Cry1A.105 insecticidal proteins. Sci. Rep. 8, 7255. 10.1038/s41598-018-25491-9 29740041 PMC5940765

[B67] Francis S. (2002). Sugar beet powdery mildew (*Erysiphe betae*). Mol. Plant Pathol. 3 (3), 119–124. 10.1046/j.1364-3703.2002.00103.x 20569317

[B68] Francis S. A. Luterbacher M. C. (2003). Identification and exploitation of novel disease resistance genes in sugar beet. Pest Manag. Sci. 59, 225–230. 10.1002/ps.569 12587876

[B69] Garg R. Verma M. Agrawal S. Shankar R. Majee M. Jain M. (2014). Deep transcriptome sequencing of wild halophyte rice, *Porteresia coarctata*, provides novel insights into the salinity and submergence tolerance factors. DNA Res. 21, 69–84. 10.1093/dnares/dst042 24104396 PMC3925395

[B70] Ghaemi R. Pourjam E. Safaie N. Verstraeten B. Mahmoudi S. B. Mehrabi R. (2020). Molecular insights into the compatible and incompatible interactions between sugar beet and the beet cyst nematode. BMC Plant Biol. 20, 483. 10.1186/s12870-020-02706-8 33092522 PMC7583174

[B71] Ghaffari H. Tadayon M. R. Bahador M. Razmjoo J. (2022). Biochemical and yield response of sugar beet to drought stress and foliar application of vermicompost tea. Plant Stress 5, 100087. 10.1016/j.stress.2022.100087

[B72] Ghouri M. Z. Munawar N. Aftab S. O. Ahmad A. (2023). “Regulation of CRISPR edited food and feed: legislation and future,” in GMOs and political stance. Editors Nawaz M. A. Golokhvast K. S. Chung G. Tsatsakis A. M. (Apple Academic Press), 261–287.

[B73] Gidner S. Lennefors B. L. Nilsson N. O. Bensefelt J. Johansson E. Gyllenspetz U. (2005). QTL mapping of BNYVV resistance from the WB41 source in sugar beet. Genome 48, 279–285. 10.1139/g04-108 15838550

[B74] Gleditzsch D. Pausch P. Muller-Esparza H. Ozcan A. Guo X. Bange G. (2019). PAM identification by CRISPR-Cas effector complexes: diversified mechanisms and structures. RNA Biol. 16 (4), 504–517. 10.1080/15476286.2018.1504546 30109815 PMC6546366

[B75] Gootenberg J. S. Abudayyeh O. O. Kellner M. J. Joung J. Collins J. J. Zhang F. (2018). Multiplexed and portable nucleic acid detection platform with Cas13, Cas12a and Csm6. Sci. 80 (360), 439–444. 10.1126/science.aaq0179 PMC5961727 29449508

[B76] Gootenberg J. S. Abudayyeh O. O. Lee J. W. Essletzbichler P. Dy A. J. Joung J. (2017). Nucleic acid detection with CRISPR- Cas13a/C2c2. Sci. 80 (356), 438–442. 10.1126/science.aam9321 PMC5526198 28408723

[B77] Greco N. D’Addabbo T. Brandonisio A. Elia F. (1993). Damage to Italian crops caused by cyst forming nematodes. J. Nematol. 25, 836–842. PMCID: PMC2619465. 19279850 PMC2619465

[B78] Greger M. Ögren E. (1991). Direct and indirect effects of Cd^2+^ on photosynthesis in sugar beet (*Beta vulgaris*). Physiol. Plant 83, 129–135. 10.1111/j.1399-3054.1991.tb01291.x

[B79] Grimmer M. K. Bean K. M. R. Asher M. J. C. (2007). Mapping of five resistance genes to sugar-beet powdery mildew using AFLP and anchored SNP markers. Theor. Appl. Genet. 115 (1), 67–75. 10.1007/s00122-007-0541-1 17426954

[B80] Grujicic G. (1958). *Heterodera schachtii* Shmidt a beet nematode in our country. Plant Prot. 49 (50), 167–174.

[B81] Guo M. Liu J. H. Ma X. Luo D. X. Gong Z. H. Lu M. H. (2016). The plant heat stress transcription factors (HSFs): structure, regulation, and function in response to abiotic stresses. Front. Plant Sci. 7, 114. 10.3389/fpls.2016.00114 26904076 PMC4746267

[B82] Gupta N. Reddy K. K. Bhattacharya D. Chakraborty S. (2021). Plant responses to geminivirus infection: guardians of the plant immunity. *Virol.* J. 18, 143. 10.1186/s12985-021-01612-1 34243802 PMC8268416

[B83] Haque A. F. M. Tasnim J. El-Shehawi A. M. Rahman M. A. Parvez M. S. Ahmed M. B. (2021). The Cd-induced morphological and photosynthetic disruption is related to the reduced Fe status and increased oxidative injuries in sugar beet. Plant Physio. Biochem. 166, 448–458. 10.1016/j.plaphy.2021.06.020 34161881

[B84] Haque E. Taniguchi H. Hassan M. Bhowmik P. Karim M. R. Smiech M. (2018). Application of CRISPR/Cas9 genome editing technology for the improvement of crops cultivated in tropical climates: recent progress, prospects, and challenges. Front. Plant Sci. 9, 617. 10.3389/fpls.2018.00617 29868073 PMC5952327

[B85] Harveson R. M. (2013). Cercospora leaf spot of sugar beet. Extension bulletin Available at: https://extensionpublications.unl.edu/assets/pdf/g1753.pdf.

[B86] Haurwitz R. E. Jinek M. Wiedenheft B. Zhou K. Doudna J. A. (2010). Sequence-and structure-specific RNA processing by a CRISPR endonuclease. Sci 329 (5997), 1355–1358. 10.1126/science.1192272 PMC3133607 20829488

[B87] Hennig L. (2012). Plant gene regulation in response to abiotic stress. Biochim. Biophys. Acta. Gen. Subj. 1819 (2), 85. 10.1016/j.bbagrm.2012.01.005 22309844

[B304] Hernández-Rodríguez C. S. Hernández-Martinez P. Rie J. V. Escriche B. Ferre J. (2013). Shared midgut binding sites for Cry1A.105, Cry1Aa, Cry1Ab, Cry1Ac and Cry1Fa proteins from *Bacillus thurigenesis* in two important corn pests, *Ostrinia nubilais* and *Spodoptera frugiperda* . PloS One 8 (7), e68164. 10.1371/journal.pone.0068164 23861865 PMC3702569

[B88] Herrera F. F. Domené-Painenao O. Cruces J. M. (2017). The history of agroecology in Venezuela: a complex and multifocal process. Agroecol. Sustain. Food Syst. 41, 401–415. 10.1080/21683565.2017.1285842

[B89] Hoffmann C. Kluge-Severin S. (2011). Growth analysis of autumn and spring sown sugar beet. Eur. J. Agron. 34, 1–9. 10.1016/j.eja.2010.09.001

[B90] Horlbeck M. A. Gilbert L. A. Villalta J. E. Adamson B. Pak R. A. Chen Y. (2016). Compact and highly active next generation libraries for CRISPR mediated gene repression and activation. Elife 5, e19760. 10.7554/eLife.19760 27661255 PMC5094855

[B91] Hsu P. D. Lander E. S. Zhang F. (2014). Development and applications of CRISPR-Cas9 for genome engineering. Cell 157, 1262–1278. 10.1016/j.cell.2014.05.010 24906146 PMC4343198

[B92] Huanga J. Xua Y. Zuoa Y. Yanga Y. Tabashnikb B. E. Wua Y. (2020). Evaluation of five candidate receptors for three Bt toxins in the beet armyworm using CRISPR-mediated gene knockouts. Insect biochem. Mol. Biol. 121, 103361. 10.1016/j.ibmb.2020.103361 32199887

[B93] Iqbal Z. Memom A. G. Ahmad A. Iqbal M. S. (2022). Calcium mediated cold acclimation in plants: underlying signaling and molecular mechanisms. Front. Plant Sci. 13, 855559. 10.3389/fpls.2022.855559 35574126 PMC9094111

[B94] Ismail R. M. Youssef A. B. El-Assal S. E. D. Tawfik M. S. Abdallah N. A. (2020). Cloning and characterization of heat shock factor (BVHSF) from sugar beet (*Beta vulgaris* L.). Plant Arch. 20 (2), 3725–3733.

[B95] Jacob P. Hirt H. Bendahmane A. (2017). The heat-shock protein/chaperone network and multiple stress resistance. Plant Biotechnol. J. 15, 405–414. 10.1111/pbi.12659 27860233 PMC5362687

[B96] Jafari M. Norouzi P. Malboobi M. A. Ghareyazie B. Valizadeh M. Mohammadi S. A. (2009). Enhanced resistance to a lepidopteran pest in transgenic sugar beet plants expressing synthetic cry1Ab gene. Euphytica 165, 333–344. 10.1007/s10681-008-9792-4

[B97] Jalilian M. Dehdari M. Fahliani R. R. Dehnovi M. M. (2017). Study of cold tolerance of different sugar beet (*Beta vulgaris* L.) cultivars at seedling growth stage. Environ. Stresses Crop Sci. 10, Pe475–Pe490. 10.22077/ESCS.2017.616,

[B98] Jamla M. Khare T. Joshi S. Patil S. Penna S. Kumar V. (2021). Omics approaches for understanding heavy metal responses and tolerance in plants. Curr. Plant Biol. 27, 100213. 10.1016/j.cpb.2021.100213

[B99] Ji X. Zhang H. Zhang Y. Wang Y. Gao C. (2015). Establishing a CRISPR-Cas-like immune system conferring DNA virus resistance in plants. Nat. Plants 1, 15144. 10.1038/nplants.2015.144 27251395

[B100] Jiang J. Ma S. Ye N. Jiang M. Cao J. Zhang J. (2017). WRKY transcription factors in plant responses to stresses. J. Integr. Plant Biol. 59 (2), 86–101. 10.1111/jipb.12513 27995748

[B101] Jiang N. M. Zhang C. Liu J. Y. Guo Z. H. Zhang Z. Y. Han C. G. (2019). Development of Beet necrotic yellow vein virus-based vectors for multiple-gene expression and guide RNA delivery in plant genome editing. Plant Biotech. J. 17, 1302–1315. 10.1111/pbi.13055 PMC6576094 30565826

[B302] Jiang S. Y. Ramamoorthy R. Bhalla R. Luan H. F. Venkatesh P. N. Cai M. (2008). Genome-wide survey of the RIP domain family in *Oryza sativa* and their expression profiles under various abiotic and biotic stresses. Plant Mol. Biol. 67, 603–614. 10.1007/s11103-008-9342-4 18493723

[B102] Jiang W. Bikard D. Cox D. Zhang F. Marraffini L. A. (2013). RNA-guided editing of bacterial genomes using CRISPR-Cas systems. Nat. Biotechnol. 31 (3), 233–239. 10.1038/nbt.2508 23360965 PMC3748948

[B103] Jiang Y. Deyholos M. K. (2008). Functional characterization of *Arabidopsis* NaCl-inducible WRKY25 and WRKY33 transcription factors in abiotic stresses. Plant Mol. Biol. 69, 91–105. 10.1007/s11103-008-9408-3 18839316

[B104] Jiao C. Sharma S. Dugar G. Peeck N. L. Bischler T. Wimmer F. (2021). Noncanonical crRNAs derived from host transcripts enable multiplexable RNA detection by Cas9. Sci. 80 (372), 941–948. 10.1126/science.abe7106 PMC8224270 33906967

[B105] Jin L. Wang J. Guan F. Zhang J. Yu S. Liu S. (2018). Dominant point mutation in a tetraspanin gene associated with field-evolved resistance of cotton bollworm to transgenic Bt cotton. *Proc. Natl. Acad. Sci*. U. S. A. 115, 11760–11765. 10.1073/pnas.1812138115 30381456 PMC6243265

[B106] Jin M. Tao J. Li Q. Cheng Y. Sun X. Wu K. (2021). Genome editing of the SfABCC2 gene confers resistance to Cry1F toxin from *Bacillus thuringiensis* in *Spodoptera frugiperda* . J. Integr. Agric. 20, 815–820. 10.1016/S2095-3119(19)62772-3

[B107] Jin M. Yang Y. Shan Y. Chakrabarty S. Cheng Y. Soberon M. (2020). Two ABC transporters are differentially involved in the toxicity of two *Bacillus thuringiensis* Cry1 toxins to the invasive crop-pest *Spodoptera frugiperda* (J. E. Smith). Pest Manag. Sci. 77, 1492–1501. 10.1002/ps.6170 33145907

[B108] Jinek M. Chylinski K. Fonfara I. Hauer M. Doudna J. A. Charpentier E. (2012). A programmable dual-RNA–guided DNA endonuclease in adaptive bacterial immunity. Sci. 337 (6096), 816–821. 10.1126/science.1225829 PMC6286148 22745249

[B109] Johansson E. (1985). Rhizomania in sugar beet-a threat to beet growing that can be overcome by plant breeding. Sveriges Utsädesförenings Tidskr. 95, 115–121.

[B110] Joshi I. Kumar A. Kohli D. Singh A. K. Sirohi A. Subramaniam K. (2020). Conferring root-knot nematode resistance *via* host-delivered RNAi-mediated silencing of four Mi-msp genes in *Arabidopsis* . Plant Sci. 298, 110592. 10.1016/j.plantsci.2020.110592 32771150

[B111] Jyoti K. Meenakshi F. Neetika M. (2023). CRISPR/Cas9: an evolutionary approach towards crops amelioration. Int. J. Life Sci. 12 (1), 44–61. 10.5958/2319-1198.2023.00004.0

[B112] Kalaitzandonakes N. Willig C. Zahringer K. (2022). The economics and policy of genome editing in crop improvement. Plant Genome 16 (2), e20248. 10.1002/tpg2.20248 36321718 PMC12807197

[B113] Karim S. Riazuddin S. Gould F. Dean D. H. (2000). Determination of receptor binding properties of *Bacillus thuringiensis* delta-endotoxins to cotton bollworm (*Helicoverpa zea*) and pink bollworm (*Pectinophora gossypiella*) midgut brush border membrane vesicles. Pestic. Biochem. Physiol. 67, 198–216. 10.1006/pest.2000.2491

[B114] Karmakar S. Das P. Panda D. Xie K. Baig M. J. Molla K. A. (2022). A detailed landscape of CRISPR Cas mediated plant disease and pest management. Plant Sci. 323, 111376. 10.1016/j.plantsci.2022.111376 35835393

[B115] Keller I. Müdsam C. Martins Rodrigues C. Kischka D. Zierer W. Sonnewald U. (2021). Cold-triggered induction of ROS- and raffinose metabolism in freezing-sensitive taproot tissue of sugar beet. Front. Plant Sci. 12, 715767. 10.3389/fpls.2021.715767 34539707 PMC8446674

[B116] Khadiza K. Khan R. A. H. Park J. I. Nath U. K. Kimm K. B. Nou I. S. (2017). Molecular characterization and expression profiling of tomato GRF transcription factor family genes in response to abiotic stresses and phytohormones. Int. J. Mole. Sci. 18 (5), 1056. 10.3390/ijms18051056 PMC5454968 28505092

[B117] Khan S. A. Reichelt M. Heckel D. G. (2017). Functional analysis of the ABCs of eye color in *Helicoverpa armigera* with CRISPR/Cas9-induced mutations. Sci. Rep. 7, 40025. 10.1038/srep40025 28053351 PMC5214861

[B118] Khatodia S. Bhatotia K. Tuteja N. (2017). Development of CRISPR/Cas9 mediated virus resistance in agriculturally important crops. Bioengineered 8 (3), 274–279. 10.1080/21655979.2017.1297347 28581909 PMC5470520

[B119] Kim S. T. Choi M. Bae S. J. Kim J. S. (2021). The functional association of acqos/victr with salt stress resistance in *Arabidopsis thaliana* was confirmed by CRISPR-mediated mutagenesis. Int. J. Mol. Sci. 22, 11389. 10.3390/ijms222111389 34768819 PMC8583979

[B120] Kimoto Y. Shimamoto Y. (2001). Difference in toxicity to larvae of cabbage armyworm between transgenic sugar beet lines with Cry1Ab and Cry1AC. Proc. J. Soc. Sugar Beet Technol. 43, 20–23.

[B121] Kimoto Y. Shimamoto Y. (2002). Differences in toxicity to larvae of cabbage armyworm between transgenic sugar beet lines with cry I A(b) and cry I C. Proc. J. Soc. Sugar Beet Technol. 43, 20–23.

[B122] Kito K. Yamane K. Yamamori T. Matsuhira H. Tanaka Y. Takabe T. (2018). Isolation, functional characterization and stress responses of raffinose synthase genes in sugar beet. J. Plant Biochem. Biotechnol. 27, 36–45. 10.1007/s13562-017-0413-y

[B123] Kocak D. Gersbach C. (2018). From CRISPR scissors to virus sensors. Nature 557 (7704), 168–169. 10.1038/d41586-018-04975-8 29730672

[B124] Koutroumpa F. A. Monsempes C. Francois M. C. Cian A. D. Royer C. Concordet J. P. (2016). Heritable genome editing with CRISPR/Cas9 induces anosmia in a crop pest moth. Sci. Rep. 6, 29620. 10.1038/srep29620 27403935 PMC4940732

[B125] Kumar A. Harloff H. J. Melzer S. Leineweber J. Defant B. Jung C. (2021). A rhomboid-like protease gene from an interspecies translocation confers resistance to cyst nematodes. New Phytol. 231, 801–813. 10.1111/nph.17394 33866563

[B126] Kumar A. Yang F. Goddard L. Schubert S. (2004). Differing trends in the tropical surface temperatures and precipitation over land and oceans. J. Clim. 17, 653–664. 10.1175/1520-0442(2004)017<0653:DTITTS>2.0.CO;2

[B127] Kumar K. Gambhir G. Dass A. Tripathi A. K. Singh A. Jha A. K. (2020). Genetically modified crops: current status and future prospects. Planta 251, 91. 10.1007/s00425-020-03372-8 32236850

[B128] Lan T. Zheng Y. Su Z. Yu S. Song H. Zheng X. (2019). OsSPL10, a SBP-Box gene, plays a dual role in salt tolerance and trichome formation in rice (*Oryza sativa* L.). G3 Genes, Genomes, Genet. 9 (12), 4107–4114. 10.1534/g3.119.400700 PMC6893181 31611344

[B129] Larbi A. Morales F. Abadía A. Gogorcena Y. Lucena J. Abadía J. (2002). Effects of Cd and Pb in sugar beet plants grown in nutrient solution: induced Fe deficiency and growth inhibition. Funct. Plant Biol. 29, 1453–1464. 10.1071/FP02090 32688745

[B130] La Russa M. F. Qi L. S. (2015). The new state of the art: cas9 for gene activation and repression. Mol. Cell Biol. 35 (22), 3800–3809. 10.1128/MCB.00512-15 26370509 PMC4609748

[B131] Lei G. Shen M. Li Z. G. Zhang B. Duan K. X. Wang N. (2020). EIN2 regulates salt stress response and interacts with a MA3 domain-containing protein ECIP1 in *Arabidopsis* . Plant Cell Environ. 34 (10), 1678–1692. 10.1111/j.1365-3040.2011.02363.x 21631530

[B132] Lein J. C. Asbach K. Tian Y. Schulte D. Li C. Koch G. (2007). Resistance gene analogues are clustered on chromosome 3 of sugar beet and cosegregate with QTL for rhizomania resistance. Genome 50, 61–71. 10.1139/g06-131 17546072

[B133] Lewellen R. T. Schrandt J. K. (2001). Inheritance of powdery mildew resistance in sugar beet derived from *Beta vulgaris* subsp maritima. Plant Dis. 85, 627–631. 10.1094/PDIS.2001.85.6.627 30823030

[B134] Lewellen R. T. Skoyen I. O. Erichsen A. W. (1987). “Breeding sugar beet for resistance to rhizomania: evaluation of host-plant reactions and selection for and inheritance of resistance,” in Proceedings of the IIRB 50th Congress, 139–156.

[B135] Li B. Liang S. Alariqi M. Wang F. Wang G. Wang Q. (2021a). The application of temperature sensitivity CRISPR/LbCpf1 (LbCas12a) mediated genome editing in allotetraploid cotton (*G. Hirsutum*) and creation of non-transgenic, gossypol-free cotton. Plant Biotechnol. J. 19 (2), 221–223. 10.1111/pbi.13470 32854160 PMC7868979

[B136] Li H. Xu Y. Xiao Y. Zhu Z. Xie X. Zhao H. (2010). Expression and functional analysis of two genes encoding transcription factors, VpWRKY1 and VpWRKY2, isolated from Chinese wild *Vitis pseudoreticulata* . Planta 232, 1325–1337. 10.1007/s00425-010-1258-y 20811906

[B137] Li J. Cui J. Dai C. Liu T. Cheng D. Luo C. (2021b). Whole-transcriptome RNA sequencing reveals the global molecular responses and cerna regulatory network of mRNAs, lncRNAs, miRNAs and circRNAs in response to salt stress in sugar beet (*Beta vulgaris*). Int. J. Mol. Sci. 22, 289. 10.3390/ijms22010289 PMC7795855 33396637

[B138] Li J. Wang Y. Wang B. Lou J. Ni P. Jin Y. (2022a). Application of CRISPR/Cas systems in the nucleic acid detection of infectious diseases. Diagnostics 12, 2455. 10.3390/diagnostics12102455 36292145 PMC9600689

[B139] Li J. Zhang M. Sun J. Mao X. Wang J. Liu H. (2020a). Heavy metal stress associated proteins in rice and *Arabidopsis*: genome-wide identification phylogenetics, duplication and expression profile analysis. Front. Gene. 11, 477. 10.3389/fgene.2020.00477 PMC7225358 32457808

[B140] Li Q. Jin M. Yu S. Cheng Y. Shan Y. Wang P. (2022b). Knockout of the ABCB1 gene increases susceptibility to emamectin benzoate, beta-cypermethrin and chlorantraniliprole in *Spodoptera frugiperda* . Insects 13, 137. 10.3390/insects13020137 35206711 PMC8875147

[B141] Li R. Zhang L. Wang L. Chen L. Zhao R. Sheng J. (2018). Reduction of tomato-plant chilling tolerance by CRISPR–Cas9 mediated SlCBF1 mutagenesis. J. Agric. Food Chem. 66, 9042–9051. 10.1021/acs.jafc.8b02177 30096237

[B142] Li S. Fu Q. Chen L. Huang W.-D. Yu D. (2011). *Arabidopsis thaliana* WRKY25, WRKY26, and WRKY33 coordinate induction of plant thermotolerance. Planta 233, 1237–1252. 10.1007/s00425-011-1375-2 21336597

[B143] Li W. Pang S. Lu Z. Jin B. (2020b). Function and mechanism of WRKY transcription factors in abiotic stress responses of plants. Plants 9, 1515. 10.3390/plants9111515 33171689 PMC7695288

[B144] Liao S. Qin X. Luo L. Han Y. Wang X. Usman B. (2019). CRISPR/Cas9-induced mutagenesis of *semi-rolled Leaf1, 2* confers curled leaf phenotype and drought tolerance by influencing protein expression patterns and ROS scavenging in rice (*Oryza sativa* L.). Agron. 9, 728. 10.3390/agronomy9110728

[B145] Liebe S. Wibberg D. Maiss E. Varrelmann M. (2020). Application of a reverse genetic system for beet necrotic yellow vein virus to study Rz1 resistance response in sugar beet. Front. Plant Sci. 10, 1703. 10.3389/fpls.2019.01703 32010172 PMC6978805

[B303] Lim C. Kang K. Shim Y. Yoo S. C. Paek N. C. (2022). Inactivating transcription factor OsWRKY5 enhances drought tolerance through abscisic acid signaling pathways. Plant Physiol. 188 (4), 1900–1916. 10.1093/plphys/kiab492 34718775 PMC8968288

[B146] Lin J. S. Huang X. X. Li Q. Cao Y. Bao Y. Meng X. F. (2016). UDP glycosyl transferase 72B1 catalyzes the glucose conjugation of monolignols and is essential for the normal cell wall lignification in *Arabidopsis thaliana* . Plant J. 88, 26–42. 10.1111/tpj.13229 27273756

[B147] Liu D. Gao Z. Li J. Yao Q. Tan W. Xing W. (2022). Effects of cadmium stress on the morphology, physiology, cellular ultrastructure, and BvHIPP24 gene expression of sugar beet (*Beta vulgaris* L.). Int. J. Phytoremediation 25 (4), 455–465. 10.1080/15226514.2022.2090496 35771710

[B148] Lombardo L. Grando M. S. (2020). Genetically modified plants for nutritionally improved food: a promise kept? Food Rev. Int. 36, 58–76. 10.1080/87559129.2019.1613664

[B149] Lou D. Wang H. Liang G. Yu D. (2017). OsSAPK2 confers abscisic acid sensitivity and tolerance to drought stress in rice. Front. Plant Sci. 8, 993. 10.3389/fpls.2017.00993 28659944 PMC5468418

[B150] Lu H. P. Liu S. M. Xu S. L. Chen W.-Y. Zhou X. Tan Y.-Y. (2017). CRISPR-S: an active interference element for a rapid and inexpensive selection of genome-edited, transgene-free rice plants. Plant Biotechnol. J. 15 (11), 1371–1373. 10.1111/pbi.12788 28688132 PMC5633759

[B151] Lu H. P. Luo T. Fu H. W. Wang L. Tan Y. Y. Huang J. Z. (2018). Resistance of rice to insect pests mediated by suppression of serotonin biosynthesis. Nat. Plants 4, 338–344. 10.1038/s41477-018-0152-7 29735983

[B152] Luterbacher M. C. Smith J. M. Asher M. J. C. (1998). Sources of disease resistance in wild Beta germplasm. Asp. Appl. Biol. 52, 423–430.

[B153] Luterbacher M. C. Smith J. M. Asher M. J. C. Frese L. (2000). Disease resistance in collections of Beta species. J. Sugar Beet Res. 37, 39–47. 10.5274/jsbr.37.3.39

[B154] Lytvyn D. I. Syvura V. V. Kurylo V. V. Olenieva V. D. Yemets A. I. Blume Y. B. (2014). Creation of transgenic sugar beet lines expressing insect pest resistance genes cry1C and cry2A. Cytol. Genet. 48 (2), 69–75. 10.3103/S0095452714020078 24818505

[B155] Lyu Y.-S. Cao L.-M. Huang W.-Q. Liu J.-X. Lu H.-P. (2022). Disruption of three polyamine uptake transporter genes in rice by CRISPR/Cas9 gene editing confers tolerance to herbicide paraquat. aBIOTECH 3 (2), 140–145. 10.1007/s42994-022-00075-4 36304519 PMC9590464

[B156] Majumdar R. Galewski P. J. Eujayl I. Minocha R. Vincill E. Strausbaugh C. A. (2022). Regulatory roles of small non-coding RNAs in sugar beet resistance against beet curly top virus. Front. Plant Sci. 12, 780877. 10.3389/fpls.2021.780877 35082811 PMC8786109

[B157] Mall A. K. Misra V. SanteshwariPathak A. D. Srivastava S. (2022b). Sugar beet cultivation in India: prospects for bioethanol production and value-added co-products. Sugar Tech. 23, 1218–1234. 10.1007/s12355-021-01007-0 PMC8261398 34248307

[B158] Mall A. K. Misra V. Srivastava S. Pathak A. D. (2022a). “India’s sugar beet seed technology and production,” in Sugar beet cultivation, management and processing. Editors Misra V. Srivastava S. Mall A. K. (Springer Nature Singapore Pte Ltd), 121–129. 10.1007/978-981-19-2730-0_7

[B159] Mallapaty S. (2022). China’s approval of gene-edited crops energizes researchers. Nature 602, 559–560. 10.1038/d41586-022-00395-x 35145296

[B160] Mao Y. Botella J. R. Liu Y. Zhu J.-K. (2019). Gene editing in plants: progress and challenges. Natl. Sci. Rev. 6 (3), 421–437. 10.1093/nsr/nwz005 34691892 PMC8291443

[B161] McCarty N. S. Graham A. E. Studena L. Ledesma-Amaro R. (2020). Multiplexed CRISPR technologies for gene editing and transcriptional regulation. Nat. Commun. 11, 1281. 10.1038/s41467-020-15053-x 32152313 PMC7062760

[B162] Miao C. Xiao L. Hua K. Zou C. Zhao Y. Bressan R. A. (2018). Mutations in a subfamily of abscisic acid receptor genes promote rice growth and productivity. Proc. Natl. Acad. Sci. U.S.A. 115, 6058–6063. 10.1073/pnas.1804774115 29784797 PMC6003368

[B163] Mickelbart M. V. Hasegawa P. M. Bailey-Serres J. (2015). Genetic mechanisms of abiotic stress tolerance that translate to crop yield stability. Nat. Rev. Genet. 16, 237–251. 10.1038/nrg3901 25752530

[B164] Misra V. Mall A. K. Kumar A. Srivastava S. Pathak A. D. (2020a). Identification of two new *Alternaria* isolates on sugar beet (*Beta vulgaris* L.) plants in Lucknow, India. Arch. Phytopathol. Plant Prot. 54, 164–176. 10.1080/03235408.2020.1824378

[B165] Misra V. Mall A. K. Kumar M. Singh D. Pathak A. D. (2018). “Sugar beet crop: asset for farmers in enhancing income India international science festival,” in Theme frontier areas in science (book 3) held at indra gandhi pratishthan (Lucknow), 43. Abstract No. 43.

[B166] Misra V. Mall A. K. Pathak A. D. (2020b). “Sugar beet: a sustainable crop for saline environment,” in Agronomic crops. Editor Hassanzuman M. (Springer Nature Singapore Pvt. Ltd), 49–61. 10.1007/978-981-15-0025-1_4

[B167] Misra V. Srivastava S. Mall A. K. (2022a). Sugar beet cultivation, management and processing. Springer Nature Singapore Pte Ltd, 1–1005. 10.1007/978-981-19-2730-0

[B168] Misra V. Srivastava S. Mall A. K. Srivastava S. (2022c). “Foliar sugar beet diseases and their management approaches in India,” in Sugar beet cultivation, management and processing. Editors Misra V. Srivastava S. Mall A. K. (Springer Nature Singapore Pte Ltd), 541–564. 10.1007/978-981-19-2730-0_26

[B169] Mo W. Tang W. Du Y. Jing Y. Bu Q. Lin R. (2020). Phytochrome interacting Factor-Like14 and Slender Rice1 interaction controls seedling growth under salt stress. Plant Physiol. 184, 506–517. 10.1104/PP.20.00024 32581115 PMC7479912

[B170] Moliterni V. M. Paris R. Onofri C. Orrù L. Cattivelli L. Pacifico D. (2015). Early transcriptional changes in *Beta vulgaris* in response to low temperature. Planta 242, 187–201. 10.1007/s00425-015-2299-z 25893871

[B171] Monteiro F. Frese L. Castro S. Duarte M. C. Paulo O. S. Louriro J. (2018). Genetic and genomic tools to asssist sugar beet improvement: the value of the crop wild relatives. Front. Plant Sci. 9, 74. 10.3389/fpls.2018.00074 29467772 PMC5808244

[B172] Moore C. E. Meacham-Hensold K. Lemonnier P. Slattery R. A. Benjamin C. Bernacchi C. J. (2021). The effect of increasing temperature on crop photosynthesis: from enzymes to ecosystems. J. Exp. Bot. 72 (8), 2822–2844. 10.1093/jxb/erab090 33619527 PMC8023210

[B173] Mulet J. M. (2022). “Shaping the sugar beet of tomorrow: current advances in Sugar beet biotechnology and new breeding techniques,” in Sugar beet cultivation, management and processing. Editors Misra V. Srivastava S. Mall A. K. (Springer Nature Singapore Pte Ltd), 49–74. 10.1007/978-981-19-2730-0_4

[B174] Murakami Y. Tsuyama M. Kobayashi Y. Kodama H. Iba K. (2000). Trienoic fatty acids and plant tolerance of high temperature. Sci 287, 476–479. 10.1126/science.287.5452.476 10642547

[B175] Mutasa-Gottgens E. S. Chwarszczynska D. M. Halsey K. Asher M. J. C. (2000). Specific polyclonal antibodies for the obligate plant parasite *Polymyxa*—a targeted recombinant DNA approach. Plant Pathol. 49, 276–287. 10.1046/j.1365-3059.2000.00446.x

[B176] Nandy S. Pathak B. Zhao S. Srivastava V. (2019). Heat-shock-inducible CRISPR/Cas9 system generates heritable mutations in rice. Plant Direct 3 (5), e00145. 10.1002/pld3.145 31404128 PMC6603394

[B177] Nawaz G. Han Y. Usman B. Liu F. Qin B. Li R. (2019). Knockout of OsPRP1, a gene encoding proline-rich protein, confers enhanced cold sensitivity in rice (*Oryza sativa* L.) at the seedling stage. 3 Biotech. 9 (7), 254. 10.1007/s13205-019-1787-4 PMC6555840 31192079

[B178] Neher O. T. Gallian J. J. (2013). Powdery mildew on sugar beet: importance, identification and control, 643. A Pacific Northwest Extension Publication, 1–6. PNW.

[B179] Nielsen K. K. Bojsen K. Roepstorff P. Mikkelsen J. D. (1994). A hydroxyproline-containing class IV chitinase of sugar beet is glycosylated with xylose. Plant Mole. Biol. 25 (2), 241–257. 10.1007/bf00023241 8018873

[B180] Nielsen K. K. Mikkelsen J. D. Kragh K. M. Bojsen K. (1993). An acidic class III chitinase in sugar beet: induction by *Cercospora beticola*, characterization, and expression in transgenic tobacco plants. Mole. Plant-Microbe Interact. 6 (4), 495–506. 10.1094/mpmi-6-495 8400378

[B181] Nieves-Cordones M. Mohamed S. Tanoi K. Kobayashi N. I. Takagi K. Vernet A. (2017). Production of low-Cs^+^ rice plants by inactivation of the K^+^ transporter of HAK 1 with the CRISPR-Cas system. Plant J. 92 (1), 43–56. 10.1111/tpj.13632 28670755

[B182] Ober E. S. Rajabi A. (2010). Abiotic stress in sugar beet. Sugar Tech. 12 (3-4), 294–298. 10.1007/s12355-010-0035-3

[B183] Ogata T. Ishizaki T. Fujita M. Fujita Y. (2020). CRISPR/Cas9-targeted mutagenesis of *OsERA1* confers enhanced responses to abscisic acid and drought stress and increased primary root growth under non-stressed conditions in rice. PloS One 15, e0243376. 10.1371/journal.pone.0243376 33270810 PMC7714338

[B184] Orduz S. Diaz T. Restrepo N. Patiño M. M. Tamayo M. C. (1996). Biochemical, immunological and toxicological characteristics of the crystal proteins of *Bacillus thuringiensis* sub sp. modelling. Mem. Inst. Oswaldo Cruz 91, 231–237. 10.1590/s0074-02761996000200020 8736096

[B185] Osakabe Y. Watanabe T. Sugano S. S. Ueta R. Ishihara R. Shinozaki K. (2016). Optimization of CRISPR/Cas9 genome editing to modify abiotic stress responses in plants. Sci. Rep. 6 (1), 26685. 10.1038/srep26685 27226176 PMC4880914

[B186] Owen F. W. Ryser G. K. (1942). Some Mendelian characters in *Beta vulgaris* L. and linkages observed in the Y-R-B group. J. Agric. Res. 65, 135–171.

[B187] Pankaj Y. K. Kumar V. (2023). “CRISPR/CAS: the Beginning of a new era in crop improvement,”. Advanced crop improvement. Editors Raina A. Wani M. R. Laskar R. A. Tomlekova N. Khan S. (Cham: Springer), 1, 489–505. 10.1007/978-3-031-28146-4_17

[B188] Pardee K. Green A. A. Takahashi M. K. Braff D. Lambert G. Lee J. W. (2016). Rapid, low-cost detection of Zika virus using programmable biomolecular components. Cell 165 (5), 1255–1266. 10.1016/j.cell.2016.04.059 27160350

[B189] Park C. Y. Lee J. H. Yoo J. H. Moon B. C. Choi M. S. Kang Y. H. (2005). WRKY group IId transcription factors interact with calmodulin. FEBS Lett. 579, 1545–1550. 10.1016/j.febslet.2005.01.057 15733871

[B190] Park J. J. Dempewolf E. Zhang W. Wang Z. Y. (2017). RNA-guided transcriptional activation *via* CRISPR/dCas9 mimics overexpression phenotypes in *Arabidopsis* . PloS One 12, e0179410. 10.1371/journal.pone.0179410 28622347 PMC5473554

[B191] Pattanayak S. Das S. Kumar S. (2023). Development of stress tolerant transgenomic traits in sugar beet through biotechnological application. J. Plant Prot. Res. 63 (1), 1–12. 10.24425/jppr.2023.144505

[B192] Pauchet Y. Bretschneider A. Augustin S. Heckel D. G. (2016). A P-glycoprotein is linked to resistance to the *Bacillus thuringiensis* Cry3Aa toxin in a leaf beetle. *Toxins* (Basel) 8, 362. 10.3390/toxins8120362 27929397 PMC5198556

[B193] Paul H. B. Henken B. Scholten O. E. Lange W. (1993). Use of zoospores of *Polymyxa betae* in screening beet seedlings for resistance to beet necrotic yellow vein virus. Neth. J. Plant Pathol. 99 (3), 151–160. 10.1007/bf03041405

[B194] Paul N. C. Park S. W. Liu H. Choi S. Ma J. MacCready J. S. (2021). Plant and fungal genome editing to enhance plant disease resistance using the CRISPR/Cas 9 system. Front. Plant Sci. 12, 700925. 10.3389/fpls.2021.700925 34447401 PMC8382960

[B195] Pferdmenges F. (2007). Dissertation beet necrotic yellow vein virus. Dissertation, 23. Cuvillier Verlag Gottingen.

[B196] Piatek A. Ali Z. Baazim H. Li L. Abulfaraj A. Al-Shareef S. (2015). RNA-guided transcriptional regulation in planta via synthetic dCas9-based transcription factors. Plant Biotechnol. J. 13 (4), 578–589. 10.1111/pbi.12284 25400128

[B197] Pidgeon J. D. Werker A. R. Jaggard K. W. Richter G. M. Lister D. H. Jones P. D. (2001). Climatic impact on the productivity of sugar beet in Europe, 1961–1995. *Agric. Meteoro.* L. 109, 27–37. 10.1016/S0168-1923(01)00254-4

[B198] Porcel R. Bustamante A. Ros R. Serrano R. Mulet Salort J. M. (2018). BvCOLD1: a novel aquaporin from sugar beet (*Beta vulgaris* L.) involved in boron homeostasis and abiotic stress. Plant Cell Environ. 41, 2844–2857. 10.1111/pce.13416 30103284

[B199] Pylypenko L. A. Kalatur K. A. (2015). Breeding and usage of sugar beet cultivars and hybrids resistant to sugar beet nematode *Heterodera schachtii* . Agric. Sci. Pract. 2, 12–22. 10.15407/agrisp2.01.012

[B200] Qi X. Tai C. Y. Wasserman B. P. (1995). Plasma membrane intrinsic proteins of *Beta vulgaris* L. Plant Physiol. 108 (1), 387–392. 10.1104/pp.108.1.387 7784509 PMC157345

[B201] Qiu Z. Kang S. He L. Zhao J. Zhang S. Hu J. (2018). The newly identified heat-stress sensitive albino 1 gene affects chloroplast development in rice. Plant Sci. 267, 168–179. 10.1016/j.plantsci.2017.11.015 29362095

[B202] Rai K. M. Ghose K. Rai A. Singh H. Srivastava R. Mendu V. (2019). “Genome engineering tools in plant synthetic biology,” in Current developments in biotechnology and bioengineering. Editors Singh S. P. Pandey A. Du G. Kumar S. (Elsevier), 47.

[B203] Ramachandran V. Weiland J. J. Bolton M. D. (2021). CRISPR-based isothermal next-generation diagnostic method for virus detection in sugarbeet. Front. Microbio. 12, 679994. 10.3389/fmicb.2021.679994 PMC8297705 34305843

[B204] Razzaq M. K. Aleem M. Mansoor S. Khan M. A. Rauf S. Iqbal S. (2021). Omics and CRISPR-Cas9 approaches for molecular insight, functional gene analysis, and stress tolerance development in crops. Int. J. Mol. Sci. 22 (3), 1292. 10.3390/ijms22031292 33525517 PMC7866018

[B205] Regev A. Keller M. Strizhov N. Sneh B. Prudovsky E. Chet I. (1996). Synergistic activity of a *Bacillus thuringiensis* d-endotoxin and a bacterial endochitinase against *Spodoptera littoralis* larvae. Appl. Environ. Microbiol. 62, 3581–3586. 10.1128/aem.62.10.3581-3586.1996 8837413 PMC168163

[B206] Reyer A. Bazihizina N. Scherzer S. Jaslan J. Schafer N. Jaslan D. (2021). Sugar beet cold induced PMT5a and STP 13 carriers are poised for taproot proton-driven plasma membrane sucrose and glucose import. bioRxiv. 10.1101/2021.09.21.461191 38602250

[B207] Roca Paixão J. F. Gillet F. X. Ribeiro T. P. Bournaud C. Lourenco-Tessutti I. T. Noriega D. D. (2019). Improved drought stress tolerance in *Arabidopsis* by CRISPR/dCas9 fusion with a histone AcetylTransferase. Sci. Rep. 9, 8080. 10.1038/s41598-019-44571-y 31147630 PMC6542788

[B208] Santeshwari Misra V. Mall A. K. Kumar D. (2020). Problems and integrated pest management strategy for *Spodoptera litura* in sugar beet in India. J. Exp. Zool. 23 (2), 1887–1890.

[B209] Santosh Kumar V. Verma R. K. Yadav S. K. Yadav P. Watts A. Rao M. V. (2020). CRISPR-Cas9 mediated genome editing of drought and salt tolerance (OsDST) gene in indica mega rice cultivar MTU1010. Physiol. Mol. Biol. Plants 26 (6), 1099–1110. 10.1007/s12298-020-00819-w 32549675 PMC7266915

[B210] Schiemann J. Robienski J. Schleissing S. Spok A. Sprink T. Wilhelm R. A. (2020). Editorial: plant genome editing - policies and governance. Front. Plant Sci. 11, 284. 10.3389/fpls.2020.00284 32218798 PMC7078341

[B211] Schmidt S. M. Belisle M. Frommer W. B. (2020). The evolving landscape around genome editing in agriculture: many countries have exempted or move to exempt forms of genome editing from GMO regulation of crop plants. EMBO Rep. 21, e50680. 10.15252/embr.202050680 32431018 PMC7271327

[B212] Scholten O. E. De Bock T. S. M. Klein-Lankhorst R. M. Lange W. (1999). Inheritance of resistance to beet necrotic yellow vein virus in Beta vulgaris conferred by a second gene for resistance. Theor. Appl. Genet. 99, 740–746. 10.1007/s001220051292 22665213

[B213] Scholten O. E. Jansen R. C. Keizer L. C. P. De Bock Th. S. M. Lange W. (1996). Major genes for resistance to beet necrotic yellow vein virus (BNYVV) in *Beta vulgaris* . Euphytica 91, 331–339. 10.1007/BF00033095

[B214] Scholten O. E. Klein-Lankhorst R. M. Esselink D. G. De Bock T. S. M. Lange W. (1997). Identification and mapping of random amplified polymorphic DNA (RAPD) markers linked to resistance against beet necrotic yellow vein virus (BNYVV) in Beta accessions. Theor. Appl. Genet. 94, 123–130. 10.1007/s001220050390 19352754

[B215] Sedighi L. Rezapanah M. Aghdam H. R. (2011). Efficacy of Bt transgenic sugar beet lines expressing *cry1Ab* gene against *Spodoptera littoralis* Boisd (Lepidoptera:]Noctuidae, Noctuidae). J. Entomol. Res. Soc. 13 (1), 61–69.

[B216] Semenova E. Jore M. M. Datsenko K. A. Semenova A. Westra E. R. Wanner B. (2011). Interference by clustered regularly interspaced short palindromic repeat (CRISPR) RNA is governed by a seed sequence. PNAS 108 (25), 10098–10103. 10.1073/pnas.1104144108 21646539 PMC3121866

[B217] Shabbir R. Singhal R. K. Mishra U. N. Chauhan J. Javed T. Hussain S. (2022). Combined abiotic stresses: challenges and potential for crop improvement. Agronomy 12 (11), 2795. 10.3390/agronomy12112795

[B218] Shah S. A. Erdmann S. Mojica F. J. Garrett R. A. (2013). Protospacer recognition motifs: mixed identities and functional diversity. RNA Biol. 10 (5), 891–899. 10.4161/rna.23764 23403393 PMC3737346

[B219] Sharma P. Dubey R. S. (2005). Lead toxicity in plants. Braz. J. Plant Physiol. 17, 35–52. 10.1590/S1677-04202005000100004

[B220] Shen C. Que Z. Xia Y. Tang N. Li D. He R. (2017). Knock out of the annexin gene OsAnn3 *via* CRISPR/Cas9-mediated genome editing decreased cold tolerance in rice. J. Plant Biol. 60, 539–547. 10.1007/s12374-016-0400-1

[B221] Shivakumara T. N. Somvanshi V. S. Phani V. Chaudhary S. Hada A. Budhwar R. (2019). *Meloidogyne incognita* (Nematoda: meloidogynidae) sterol-binding protein Mi-SBP-1 as a target for its management. Int. J. Parasitol. 49 (13-14), 1061–1073. 10.1016/j.ijpara.2019.09.002 31733196

[B222] Singh S. Kumar N. R. Maniraj R. Lakshmikanth R. Rao K. Y. S. Muralimohan N. (2018). Expression of Cry2Aa, a *Bacillus thuringiensis* insecticidal protein in transgenic pigeon pea confers resistance to Gram pod borer, *Helicoverpa armigera* . Sci. Rep. 8, 8820. 10.1038/s41598-018-26358-9 29891840 PMC5995972

[B223] Singh S. Parihar P. Singh R. Singh V. P. Prasad S. M. (2016). Heavy metal tolerance in plants: role of transcriptomics, proteomics, metabolomics and ionomics. Front. Plant Sci. 6, 1143. 10.3389/fpls.2015.01143 26904030 PMC4744854

[B224] Smigocki A. C. Ivic S. D. Wilson D. Wozniak C. A. Campbell L. Dregseth R. (2003). “Molecular approaches for control of the sugar beet root maggot,” in 1st joint IIRB-ASSBT Congress, San Antonio, USA, 26th Feb-1st March 2003, 416–428.

[B225] Smith G. A. Ruppel E. G. (1973). Association of *Cercospora* leaf spot, gross sucrose, percentage sucrose, and root weight in sugar beet. Can. J. Plant Sci. 53, 695–696. 10.4141/cjps73-136

[B226] Stevanato P. Chiodi C. Broccanello C. Concheri G. Biancardi E. Pavli O. (2019). Sustainability of the sugar beet crop. Sugar Tech. 21, 703–716. 10.1007/s12355-019-00734-9

[B227] Stewart S. D. Adamczyk J. J. Kinghten K. S. Davis F. M. (2001). Impact of *Bt* cotton expressing one or two insecticidal proteins of *Bacillus thuringiensis* Berliner on growth and survival of Noctuidae (Lepidoptera) larvae. J. Econ. Entomol. 94 (3), 752–760. 10.1603/0022-0493-94.3.752 11425033

[B228] Stokstad E. (2021). U.K. set to loosen rules for gene-edited crops and animals. Sci. News Sci. Insid. 10.1126/science.abj6955

[B229] Strausbaugh C. A. Eujayl I. A. Wintermantel W. M. (2017). Beet curly top virus strains associated with sugar beet in Idaho, Oregon, and a Western U.S. Collection. Plant Dis. 101 (8), 1373–1382. 10.1094/PDIS-03-17-0381-RE 30678603

[B230] Strausbaugh C. A. Wenninger E. J. Eujayl I. A. (2012). Management of severe curly top in sugar beet with insecticides. Plant Dis. 96, 1159–1164. 10.1094/PDIS-01-12-0106-RE 30727055

[B231] Sun D. Guo Z. Liu Y. Zhang Y. (2017). Progress and prospects of CRISPR/Cas systems in insects and other arthropods. Front. Physiol. 8, 608. 10.3389/fphys.2017.00608 28932198 PMC5592444

[B232] Sun J. Hu W. Zhou R. Wang L. Wang X. Wang Q. (2014). The *Brachypodium distachyon* BdWRKY36 gene confers tolerance to drought stress in transgenic tobacco plants. Plant Cell Rep. 34, 23–35. 10.1007/s00299-014-1684-6 25224555

[B233] Taguchi K. Kubo T. Takahashi H. Abe H. (2011). Identification and precise mapping of resistant QTLs of *Cercospora* leaf spot resistance in sugar beet (*Beta vulgaris* L. *G3 (Bethesda*) 1 (4), 283–291. 10.1534/g3.111.000513 22384339 PMC3276142

[B234] Taguchi K. Okazaki K. Takashi H. Kubo T. Mikami T. (2010). Molecular mapping of a gene conferring resistance to *Aphanomyces* root rot (black rot) in sugar beet (*Beta vulgaris* L). Euphytica 173, 408–418. 10.1007/s10681-010-0153-8

[B235] Takenaka S. Tamagake H. (2009). Foliar spray of a cell wall protein fraction from the biocontrol agent *Pythium oligandrum* induces defence-related genes and increases resistance against *Cercospora* leaf spot in sugar beet. J. Gen. Plant Pathol. 75 (5), 340–348. 10.1007/s10327-009-0186-9

[B236] Tan W. Li K. Liu D. Xing W. (2023). Cercospora leaf spot disease of sugar beet. Plant Signal Behav. 18 (1), 2214765. 10.1080/15592324.2023.2214765 37209061 PMC10202088

[B237] Tang X. Lowder L. G. Zhang T. Malzahn A. A. Zheng X. Voytas D. F. (2017). Correction: a CRISPR–Cpf1 system for efficient genome editing and transcriptional repression in plants. Nat. Plants 3 (7), 17103. 10.1038/nplants.2017.103 28628131

[B238] Thakur M. Praveen S. Divte P. R. Mitra R. Kumar M. Gupta C. K. (2022). Metal tolerance in plants: molecular and physicochemical interface determines the "not so heavy effect" of heavy metals. Chemosphere 287 (1), 131957. 10.1016/j.chemosphere.2021.131957 34450367

[B239] Thomas M. Parry-Smith D. Iyer V. (2019). Best practice for CRISPR design using current tools and resources. Methods 164, 3–17. 10.1016/j.ymeth.2019.05.019 31152780

[B240] Thurau T. Kifle S. Jung C. Cai D. (2003). The promoter of the nematode resistance gene Hs1^pro-1^ activates a nematode responsive and feeding site-specific gene expression in sugar beet (*Beta vulgaris* L.) and *Arabidopsis thaliana* . Plant Mol. Biol. 52 (3), 643–660. 10.1023/a:1024887516581 12956533

[B241] Trela Z. Burdach Z. Przestalski S. Karcz W. (2012). Effect of trimethyllead chloride on slowly activating (SV) channels in red beet (*Beta vulgaris* L.) taproots. Comptes Rendus Biol. 335, 722–730. 10.1016/j.crvi.2012.11.004 23312295

[B242] Uphoff H. Wricke G. (1992). Random amplified polymorphic DNA (RAPD) markers in sugar beet (*Beta vulgaris* L): mapping the genes for nematode resistance and hypocotyl colour. Plant Breed. 109, 168–171. 10.1111/j.1439-0523.1992.tb00167.x

[B243] Vinson C. C. Mota A. P. Z. Porto B. N. Oliveira T. N. Sampaio I. Lacerda A. L. (2020). Characterization of raffinose metabolism genes uncovers a wild Arachis galactinol synthase conferring tolerance to abiotic stresses. Sci. Rep. 10, 15258. 10.1038/s41598-020-72191-4 32943670 PMC7498584

[B244] Wan L. Wang Z. Tang M. Hong D. Sun Y. Ren J. (2021). CRISPR-Cas9 gene editing for fruit and vegetable crops: strategies and prospects. Hortic 7, 193. 10.3390/horticulturae7070193

[B245] Wang B. Xie G. Liu Z. He R. Han J. Huang S. (2019). Mutagenesis reveals that the OsPPa6 gene is required for enhancing the alkaline tolerance in rice. Front. Plant Sci. 10, 759. 10.3389/fpls.2019.00759 31244876 PMC6580931

[B246] Wang B. Zhong Z. Wang X. Han X. Yu D. Wang C. (2020). Knockout of the OsNAC006 transcription factor causes drought and heat sensitivity in rice. Int. J. Mol. Sci. 21, 2288. 10.3390/ijms21072288 32225072 PMC7177362

[B247] Wang F. Hou X. Tang J. Wang Z. Wang S. Jiang F. (2011). A novel cold-inducible gene from Pak-choi (*Brassica campestris ssp. chinensis*), BcWRKY46, enhances the cold, salt and dehydration stress tolerance in transgenic tobacco. Mol. Biol. Rep. 39, 4553–4564. 10.1007/s11033-011-1245-9 21938429

[B248] Wang F. Z. Chen M. X. Yu L. J. Xie L. J. Yuan L.-B. Qi H. (2017b). OsARM1, an R2R3 MYB transcription factor, is involved in regulation of the response to arsenic stress in rice. Front. Plant Sci. 8, 1868. 10.3389/fpls.2017.01868 29163593 PMC5670359

[B249] Wang G. Liang G. Wu K. Guo Y. (2005). Gene cloning and sequencing of aminopeptidase N3, a putative receptor for *Bacillus thuringiensis* insecticidal Cry1Ac toxin in *Helicoverpa armigera* (Lepidoptera: noctuidae). Eur. J. Entomol. 102, 13–19. 10.14411/eje.2005.002

[B250] Wang H. Shi Y. Wang L. Liu S. Wu S. Yang Y. (2018). CYP6AE gene cluster knockout in *Helicoverpa armigera* reveals role in detoxification of phytochemicals and insecticides. Nat. Commun. 9, 4820. 10.1038/s41467-018-07226-6 30446639 PMC6240031

[B251] Wang J. Wang H. Liu S. Liu L. Tay W. T. Walsh T. K. (2017a). CRISPR/Cas9 mediated genome editing of *Helicoverpa armigera* with mutations of an ABC transporter gene HaABCA2 confers resistance to *Bacillus thuringiensis* Cry2A toxins. Insect biochem. Mol. Biol. 87, 147–153. 10.1016/j.ibmb.2017.07.002 28705634

[B252] Wang J. Zhang H. Wang H. Zhao S. Zuo Y. Yang Y. (2016). Functional validation of cadherin as a receptor of Bt toxin Cry1Acin *Helicoverpa armigera* utilizing the CRISPR/Cas9 system. Insect biochem. Mol. Biol. 76, 11–17. 10.1016/j.ibmb.2016.06.008 27343383

[B253] Wang M. Vannozzi A. Wang G. Liang Y. H. Tornielli G. B. Zenoni S. (2014). Genome and transcriptome analysis of the grapevine (Vitis vinifera L.) WRKY gene family. Hortic. Res. 1, 14016. 10.1038/hortres.2014.16 26504535 PMC4596322

[B254] Wang S. Yi F. Qu J. (2015a). Eliminate mitochondrial diseases by gene editing in germ-line cells and embryos. Protein Cell 6, 472–475. 10.1007/s13238-015-0177-x 26081469 PMC4491053

[B255] Wang T. Xun H. Wang W. Ding X. Tian H. Hussain S. (2021b). Mutation of GmAITR genes by CRISPR/cas9 genome editing results in enhanced salinity stress tolerance in soybean. Front. Plant Sci. 12, 779598. 10.3389/fpls.2021.779598 34899806 PMC8660858

[B256] Wang W. Vinocur B. Altman A. (2003). Plant responses to drought, salinity and extreme temperatures: towards genetic engineering for stress tolerance. Planta 218, 1–14. 10.1007/s00425-003-1105-5 14513379

[B257] Wang Y. Stevanato P. Yu L. Zhao H. Sun X. Sun F. (2017). The physiological and metabolic changes in sugar beet seedlings under different levels of salt stress. J. Plant Res. 130, 1079–1093. 10.1007/s10265-017-0964-y 28711996

[B258] Wang Y. Wang S. Tian Y. Wang Q. Chen S. Li H. (2021a). Functional characterization of a sugar beet BVBHLH93 transcription factor in salt stress tolerance. Int. J. Mol. Sci. 22, 3669. 10.3390/ijms22073669 33915978 PMC8037259

[B259] Wang Y. H. Chen X. E. Yang Y. Xu J. Fang G. Q. Niu C. Y. (2019). The Masc gene product controls masculinization in the black cutworm, *Agrotis ipsilon* . Insect Sci. 26 (6), 1037–1044. 10.1111/1744-7917.12635 30088858

[B260] Wang Z. Wang Y. Tong Q. Xu G. Xu M. Li H. (2021). Transcriptomic analysis of grapevine Dof transcription factor gene family in response to cold stress and functional analyses of the VaDof17d gene. Planta 253, 55. 10.1007/s00425-021-03574-8 33523295

[B261] War A. R. Paulraj M. G. Ahmad T. Buhroo A. A. Hussain B. Ignacimuthu S. (2012). Mechanisms of plant defense against insect herbivores. Plant Signal Behav. 7, 1306–1320. 10.4161/psb.21663 22895106 PMC3493419

[B262] Westra E. R. Semenova E. Datsenko K. A. Jackson R. N. Wiedenheft B. Severinov K. (2013). Type IE CRISPR-cas systems discriminate target from non-target DNA through base pairing-independent PAM recognition. PLoS Genet. 9 (9), e1003742. 10.1371/journal.pgen.1003742 24039596 PMC3764190

[B263] Wetzel V. Willems G. DarracqGalein Y. Liebe S. Varrelmann T. Varrelmann M. (2021). The *Beta vulgaris* derived resistance gene Rz2 confers broad spectrum resistance against soil borne sugar beet infecting viruses from different families by recognizing triple gene block protein 1. Mol. Plant Pathol. 22 (7), 829–842. 10.1111/mpp.13066 33951264 PMC8232027

[B264] Wilhite S. E. Elden T. C. Puizdar V. Armstrong S. Smigocki A. C. (2000). Inhibition of aspartyl and serine proteinases in the midgut of sugarbeet root maggot with proteinase inhibitors. Entomol. Exp. Appl. 97, 229–233. 10.1046/j.1570-7458.2000.00734.x

[B265] Windels C. E. (2000). Aphanomyces root rot on sugar beet. Plant Health Prog. 1 (1), 1–6. 10.1094/PHP-2000-0720-01-DG

[B266] Wu G. Q. Li Z. Q. Cao H. Wang J. L. (2019). Genome-wide identification and expression analysis of the WRKY genes in sugar beet (*Beta vulgaris* L.) under alkaline stress. Peer J. 7, e7817. 10.7717/peerj.7817 31632850 PMC6796966

[B267] Wu J. Yan G. Duan Z. Wang Z. Kang C. Guo L. (2020). Roles of the *Brassica napus* DELLA protein BnaA6. RGA, in modulating drought tolerance by interacting with the ABA signalling component BnaA10. ABF2. *Front. Plant Sci.* 11, 577. 10.3389/fpls.2020.00577 32477388 PMC7240051

[B268] Wu T. Y. Hoh K. L. Boonyaves K. Krishnamoorthi S. Urano D. (2022). Diversification of heat shock transcription factors expanded thermal stress responses during early plant evolution. Plant Cell 34 (10), 3557–3576. 10.1093/plcell/koac204 35849348 PMC9516188

[B269] Yang Y. Wang Y. H. Chen X. E. Tian D. Xu X. Li K. (2018). CRISPR/Cas9-mediated Tyrosine hydroxylase knockout resulting in larval lethality in *Agrotis ipsilon* . Insect Sci. 25 (6), 1017–1024. 10.1111/1744-7917.12647 30328670

[B270] Yerzhebayeva R. Abekova. A. Konysbekov K. Bastaubayeva S. Kabdrakhmanova A. Absattarova A. (2018). Two sugar beet chitinase genes, BvSP2 and BvSE2, analysed with SNP Amplifluor-like markers, are highly expressed after Fusarium root rot inoculations and field susceptibility trial. Peer J. 6, e5127. 10.7717/peerj.5127 29967753 PMC6026450

[B271] Yildirim K. Kavas M. Kucuk İ. S. Secgin Z. Sarac C. G. (2023). Development of highly efficient resistance to beet curly top Iran virus (Becurtovirus) in sugar beet (*B. vulgaris*) via CRISPR/Cas9 System. Int. J. Mol. Sci. 24, 6515. 10.3390/ijms24076515 37047489 PMC10095410

[B272] Yildirim K. Secgin Z. Senyer A. Can C. Kavas M. (2019). “Conferring multiple resistance to DNA viruses in plants with CRISPR/Cas9 genome editing technology,” in 1st Plant Ed Conference Plant genome editing at State of the Art, Novi Sad.

[B273] Yildrim K. Kavas M. Kaya R. Secgin Z. Can C. Sevgen I. (2022). Genome based identification of beet curly top Iran virus infecting sugar beet in Turkey and investigation of its pathogenicity by agroinfection. J. Virol. Methods 300, 114380. 10.1016/j.jviromet.2021.114380 34838538

[B274] Yin K. Gao C. Qiu J. L. (2017). Progress and prospects in plant genome editing. Nat. Plants 3, 17107. 10.1038/nplants.2017.107 28758991

[B275] Yin W. Xiao Y. Niu M. Meng W. Li L. Zhang X. (2020). ARGONAUTE2 enhances grain length and salt tolerance by activating BIG GRAIN3 to modulate cytokinin distribution in rice. Plant Cell 32, 2292–2306. 10.1105/tpc.19.00542 32409321 PMC7346564

[B276] Yolcu S. Alavilli H. Ganesh P. Asif M. Kumar M. Song K. (2021). An insight into the abiotic stress responses of cultivated beets (*Beta vulgaris* L.). Plants 11 (1), 12. 10.3390/plants11010012 35009016 PMC8747243

[B277] Yu B. Chen M. Grin I. Ma C. (2020). “Mechanisms of sugar beet response to biotic and abiotic stresses,” in Mechanisms of genome protection and repair. Editor Zharkov D. O. (Cham: Springer), 167–194. 10.1007/978-3-030-41283-8_10 32383121

[B278] Yuan Q. Metterville D. Briscoe A. D. Reppert S. M. (2007). Insect cryptochromes: gene duplication and loss define diverse ways to construct insect circadian clocks. Mol. Biol. Evol. 24 (4), 948–955. 10.1093/molbev/msm011 17244599

[B279] Zeng D. D. Yang C. C. Qin R. Alamin M. Yue E. K. Jin X. L. (2018). A guanine insert in OsBBS1 leads to early leaf senescence and salt stress sensitivity in rice (*Oryza sativa* L.). Plant Cell Rep. 37, 933–946. 10.1007/s00299-018-2280-y 29572657

[B280] Zeng Y. Wen J. Zhao W. Wang Q. Huang W. (2020). Rational improvement of rice yield and cold tolerance by editing the three genes OsPIN5b, GS3, and OsMYB30 with the CRISPR–Cas9 system. Front. Plant Sci. 10, 1663–1946. 10.3389/fpls.2019.01663 31993066 PMC6964726

[B281] Zetsche B. Gootenberg J. S. Abudayyeh O. O. Slaymaker I. M. Makarova K. S. Essletzbichler P. (2015). Cpf1 is a single RNA-guided endonuclease of a class 2 CRISPR-Cas system. Cell 163 (3), 759–771. 10.1016/j.cell.2015.09.038 26422227 PMC4638220

[B282] Zhang A. Liu Y. Wang F. Li T. Chen Z. Kong D. (2019). Enhanced rice salinity tolerance via CRISPR/Cas9-targeted mutagenesis of the *OsRR22* gene. Mol. Breed. 39, 47–10. 10.1007/s11032-019-0954-y 32803201 PMC7413041

[B283] Zhang C. L. Xu D. C. Jiang X. C. Zhou Y. Cui J. Zhang C. X. (2008). Genetic approaches to sustainable pest management in sugar beet (*Beta vulgaris* L). Ann. Appl. Biol. 152 (2), 143–156. 10.1111/j.1744-7348.2008.00228.x

[B284] Zhang J. Jin M. Yang Yliu L. Yang Y. Gómez I. Bravo A. (2020a). The cadherin protein is not involved in susceptibility to *Bacillus thuringiensis* Cry1Ab or Cry1Fa toxins in *Spodoptera frugiperda* . Toxins (Basel) 12 (6), 375. 10.3390/toxins12060375 32517191 PMC7354596

[B285] Zhang X. Long Y. Huang J. Xia J. (2020b). OsNAC45 is involved in ABA response and salt tolerance in rice. Rice 13, 79–13. 10.1186/s12284-020-00440-1 33284415 PMC7721851

[B286] Zhang Y. Guo W. Chen L. Shen X. Yang H. Fang Y. (2022). *CRISPR/Cas9-mediated targeted mutagenesis of* GmUGT *enhanced soybean resistance against leaf-chewing insects through flavonoids biosynthesis* . Front. Plant Sci. 13, 802716. 10.3389/fpls.2022.802716 35273623 PMC8902248

[B287] Zhao Y. Zhang C. Liu W. Gao W. Liu C. Song G. (2016). An alternative strategy for targeted gene replacement in plants using a dual-sgRNA/Cas9 design. Sci. Rep. 6, 23890. 10.1038/srep23890 27033976 PMC4817149

[B288] Zheng J. C. Yue X. R. Kuang W. Q. Li S. L. Tang R. Zhang Z. F. (2020). NPC1b as a novel target in controlling the cotton bollworm, *Helicoverpa armigera* . Pest Manag. Sci. 76, 2233–2242. 10.1002/ps.5761 31976620

[B289] Zhou C. Liu D. Wu P. Wang Y. Gai Z. Liu L. (2020). Transcriptome analysis of sugar beet (*Beta vulgaris* L.) in response to alkaline stress. Plant mole. Biol. 102, 645–657. 10.1007/s11103-020-00971-7 32040759

[B290] Zhu G. H. Chereddy S. C. Howell J. L. Palli S. R. (2020). Genome editing in the fall armyworm, *Spodoptera frugiperda*: multiple sgRNA/Cas9 method for identification of knockouts in one generation. Insect biochem. Mol. Biol. 122, 103373. 10.1016/j.ibmb.2020.103373 32276113

[B291] Zhu G. H. Peng Y. C. Zheng M. Y. Zhang X. Q. Sun J. B. Huang Y. (2017). CRISPR/Cas9 mediated BLOS2 knockout resulting in disappearance of yellow strips and white spots on the larval integument in *Spodoptera litura* . J. Insect Physiol. 103, 29–35. 10.1016/j.jinsphys.2017.09.008 28927827

[B292] Zhu G. H. Xu J. Cui Z. Dong X. T. Ye Z. F. Niu D. J. (2016). Functional characterization of SlitPBP3 in *Spodoptera litura* by CRIPSR/Cas9 mediated genome editing. Insect biochem. Mol. Biol. 75, 1–9. 10.1016/j.ibmb.2016.05.006 27192033

[B293] Zhu Z. Shi J. Cao J. He M. Wang Y. (2012). VpWRKY3, a biotic and abiotic stress-related transcription factor from the Chinese wild *Vitis pseudoreticulata* . Plant Cell Rep. 31, 2109–2120. 10.1007/s00299-012-1321-1 22847334

[B294] Zicari S. Zhang R. Kaffka S. (2019). “Sugar beet,” in Integrated processing technologies for food and agricultural by-products. Editors Pan Z. Zhang R. Zircari S. (Apple Academic Press), 331–351. 10.1016/b978-0-12-814138-0.00013-7

[B295] Zou C. Jiang W. Yu D. (2010). Male gametophyte-specific WRKY34 transcription factor mediates cold sensitivity of mature pollen in *Arabidopsis* . J. Exp. Bot. 61, 3901–3914. 10.1093/jxb/erq204 20643804 PMC2935866

[B296] Zou C. Wang Y. Wang B. Liu D. Liu L. Gai Z. (2020). Long non-coding RNAs in the alkaline stress response in sugar beet (*Beta vulgaris* L.). BMC Plant Biol. 20, 227. 10.1186/s12870-020-02437-w 32434543 PMC7241001

[B297] Zuo Y. Shi Y. Zhang F. Guan F. Zhang J. Feyereisen R. (2021). Genome mapping coupled with CRISPR gene editing reveals a P450 gene confers avermectin resistance in the beet armyworm. PLoS Genet. 17 (7), e1009680. 10.1371/journal.pgen.1009680 34252082 PMC8297932

[B298] Zuo Y. Wang H. Xu Y. Huang J. Wu S. Wu Y. (2017). CRISPR/Cas9 mediated G4946E substitution in the ryanodine receptor of *Spodoptera exigua* confers high levels of resistance to diamide insecticides. Insect biochem. Mol. Biol. 89, 79–85. 10.1016/j.ibmb.2017.09.005 28912111

[B299] Zuo Y. Xue Y. Lu W. Ma H. Chen M. Wu Y. (2020). Functional validation of nicotinic acetylcholine receptor (nAChR) α6as a target of spinosyns in *Spodoptera exigua* utilizing the CRISPR/Cas9 system. Pest Manag. Sci. 76, 2415–2422. 10.1002/ps.5782 32056365

[B300] Zuo Y. Y. Huang J. L. Wang J. Feng Y. Han T. T. Wu Y. D. (2018). Knockout of a P-glycoprotein gene increases susceptibility to abamectin and emamectin benzoate in *Spodoptera exigua* . Insect Mol. Biol. 27, 36–45. 10.1111/imb.12338 28753233

